# Microtubule binding of the human augmin complex is directly controlled by importins and Ran-GTP

**DOI:** 10.1242/jcs.261096

**Published:** 2023-06-26

**Authors:** Kseniya Ustinova, Felix Ruhnow, Maria Gili, Thomas Surrey

**Affiliations:** ^1^ Centre for Genomic Regulation (CRG), Department of Quantitative Cell Biology, The Barcelona Institute of Science and Technology (BIST), Carrer del Dr. Aiguader 88, 08003 Barcelona, Spain; ^2^ Universitat Pompeu Fabra (UPF), Barcelona, Spain; ^3^ Catalan Institution for Research and Advanced Studies (ICREA), Passeig Lluis Companys 23, Barcelona 08010, Spain

**Keywords:** HAUS complex, Microtubule nucleation, Importins, Ran, NLS, *In vitro* reconstitution

## Abstract

Mitotic spindle assembly during cell division is a highly regulated process. Ran-GTP produced around chromosomes controls the activity of a multitude of spindle assembly factors by releasing them from inhibitory interaction with importins. A major consequence of Ran-GTP regulation is the local stimulation of branched microtubule nucleation around chromosomes, which is mediated by the augmin complex (composed of the eight subunits HAUS1-HAUS8), a process that is crucially important for correct spindle assembly. However, augmin is not known to be a direct target of the Ran-GTP pathway, raising the question of how its activity is controlled. Here, we present the *in vitro* reconstitution of Ran-GTP-regulated microtubule binding of the human augmin complex. We demonstrate that importins directly bind to augmin, which prevents augmin from binding to microtubules. Ran-GTP relieves this inhibition. Therefore, the augmin complex is a direct target of the Ran-GTP pathway, suggesting that branching microtubule nucleation is directly regulated by the Ran-GTP gradient around chromosomes in dividing cells.

## Teaser

**Summary:** The HAUS complex is a direct target of the Ran-GTP pathway, suggesting that branching microtubule nucleation is directly regulated by the Ran-GTP gradient around chromosomes in dividing cells.

## INTRODUCTION

Mitotic spindle assembly and function rely on the precise spatiotemporal regulation of the nucleation of microtubules, their dynamic properties, and their spatial organization. Although centrosomes are a major source of microtubule nucleation, during cell division microtubules are also nucleated in the vicinity of chromosomes. Errors in this nucleation pathway lead to spindle defects and chromosome segregation errors, causing cancer or aneuploidy (Asteriti et al., 2010; Cimini et al., 2001). Chromatin-mediated nucleation is controlled by Ran-GTP generated at the chromosomes (Oh et al., 2016). Ran-GTP displaces inhibitory importins from nuclear localization signal (NLS)-containing proteins (Clarke and Zhang, 2008; Kalab and Heald, 2008; Kalab et al., 2002). Several crucial spindle proteins, also called spindle assembly factors (SAFs), are regulated in this manner (Ems-McClung et al., 2004; Gruss et al., 2001; Koffa et al., 2006; Ribbeck et al., 2006; Wiese et al., 2001).

Microtubule nucleation around chromosomes requires the recruitment of the major microtubule nucleator γ-tubulin ring complex (γTURC) to pre-existing microtubules by the augmin complex (sometimes also called the HAUS complex) (Moritz et al., 2000; Prosser and Pelletier, 2017). This nucleation mechanism leads to branched microtubule nucleation with freshly nucleated microtubules being oriented roughly parallel to pre-existing ones (Petry et al., 2013). However, neither augmin nor γTURC is known to interact with importins, and hence they are not thought to be direct targets of the Ran-GTP pathway.

Instead, work mostly in *Xenopus* egg extract has shown that branched microtubule nucleation is stimulated by the SAF TPX2 (Alfaro-Aco et al., 2017; Gruss et al., 2002; Petry et al., 2013), which has been proposed to stimulate nucleation by activating aurora A kinase (Bayliss et al., 2003; Eyers and Maller, 2004), which in turn may phosphorylate γTURC regulators (Pinyol et al., 2013), or by binding directly to augmin and/or γTURC (Alfaro-Aco et al., 2020). However, neither the aurora A kinase-activating nor the importin-binding part of TPX2 (Alfaro-Aco et al., 2017; Safari et al., 2021) is strictly required for Ran-GTP-dependent microtubule nucleation in *Xenopus* egg extract. Moreover, TPX2 is entirely dispensable for branched microtubule nucleation in *Drosophila* cells (Goshima, 2011; Verma and Maresca, 2019), and recent *in vitro* reconstitutions of branched microtubule nucleation with purified human (Zhang et al., 2022) and *Drosophila* (Tariq et al., 2020) proteins were performed in the absence of TPX2. This leaves the question unanswered of how the Ran-GTP pathway controls branching microtubule nucleation mediated by the augmin complex.

The augmin complex is evolutionarily conserved in vertebrates, insects, plants and fungi (Edzuka et al., 2014). The hetero-octameric nature of the complex and its importance for spindle assembly was first described for *Drosophila* cells (Goshima et al., 2008). Knockdown or depletion of augmin in cells leads to a dramatic reduction in spindle microtubule density, including in kinetochore fibers, and results in defects in spindle polarity and chromosome segregation both in *Drosophila* and in vertebrates and plants (David et al., 2019; Goshima et al., 2008; Ho et al., 2011; Lawo et al., 2009).

The eight subunits of the augmin complex have been reported to form two biochemically distinct subcomplexes, called tetramer T-II (containing subunits HAUS-2, -6, -7 and -8) and tetramer T-III (containing subunits HAUS-1, -3, -4 and -5) (Lawo et al., 2009). Recent cryo-electron microscopy and single-particle analysis studies showed that both tetramers together form an interconnected, highly flexible Y-shaped octameric complex (with a V-shaped head and a filamentous tail) (Gabel et al., 2022; Travis et al., 2023; Zupa et al., 2022). The primary microtubule-binding site has been localized to the intrinsically disordered N terminus of HAUS8 (Hsia et al., 2014; Wu et al., 2008), and a second, minor microtubule-binding site was recently located within the HAUS6 subunit (Travis et al., 2023; Zupa et al., 2022), with both binding sites located in the V-shaped head. *In vitro* experiments suggest that several augmin complexes together recruit γTURC (Zhang et al., 2022), but how the binding of augmin to microtubules is controlled remains unknown.

Here, we report the *in vitro* reconstitution of Ran-GTP-regulated microtubule binding of the recombinant human augmin complex composed of all eight full-length subunits. We show that importins directly bind the augmin complex, thereby suppressing its microtubule binding, and that Ran-GTP relieves this inhibition of microtubule binding. We identify a previously unknown NLS motif in HAUS8 that mediates importin and microtubule binding. These findings demonstrate that the augmin complex is a direct target of the Ran-GTP pathway, suggesting that branching microtubule nucleation is directly regulated by the Ran-GTP gradient in cells.

## RESULTS

### Recombinant human augmin complex

**Fig. 1.**
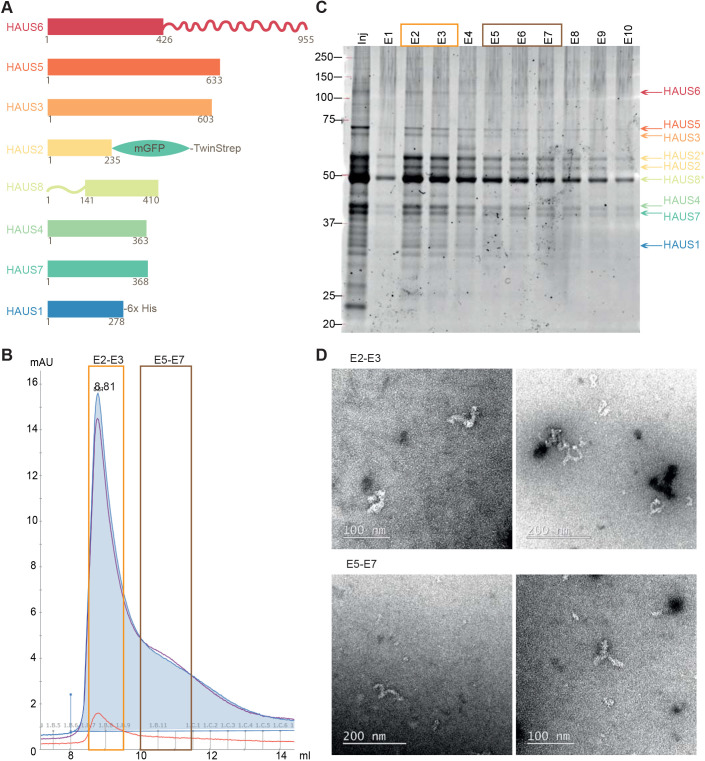
**Purification and characterization of the human augmin complex.** (A) Schematic of the eight subunits of the augmin complex. HAUS1 carries a C-terminal 3C-His_6_-tag, and HAUS2 has a C-terminal mGFP-TEV-TwinStrep tag. Numbers indicate amino acid positions. (B) Absorbance profile of the human augmin complex eluting from a Superose 6 Increase 10/30 size-exclusion chromatography column. Absorbances at 260, 280 and 455 nm are indicated in purple, blue and orange, respectively. The complete complex used for experiments in this study elutes in fractions E2–E3 (orange box). Fractions E5–E7 (brown box) contain mostly subcomplexes. mAU, the milli-absorbance unit. (C) SYPRO Ruby-stained SDS-PAGE gel of the fractions eluted from the size-exclusion chromatography column. Orange and brown boxes indicate separately pooled fractions. HAUS2* indicates HAUS2 not having undergone TEV cleavage of the affinity tag. Positions of molecular mass markers are show in kDa. (D) Negative-stain electron microscopy of the different pools: fractions E2–E3 contain the complete complex, and fractions E5–E7 also contain subcomplexes. Data in B–C are representative of three experiments. Data in D are representative of five (E2-E3) or two (E5-E7) experiments.

We expressed the human augmin complex in insect cells using a biGBac baculovirus carrying the genes of all eight augmin full-length subunits in codon-optimized form (Fig. 1A; Fig. S1A–C). HAUS2 was tagged at its C terminus with mGFP followed by a cleavable TwinStrep tag. The complex was purified by affinity (Fig. S1E) and size-exclusion chromatography. The affinity tags were partially removed during purification (Fig. 1C; HAUS2* represents the fraction of uncleaved HAUS2). Fractions eluted just after the exclusion volume of the size-exclusion column were pooled (fractions E2–E3) (Fig. 1B) and the presence of all subunits was verified by SDS-PAGE (Fig. 1C) and western blotting (Fig. S1F), yielding a purified complex similar to previously purified recombinant *Xenopus* and human augmin complexes (Gabel et al., 2022; Song et al., 2018). HAUS8 migrated as a double band (HAUS8*), as in an augmin preparation directly retrieved from cultured human cells (Zhang et al., 2022), which is due to phosphorylation, as we confirmed by mass spectrometry (Fig. S2A,B) and as observed previously (Hornbeck et al., 2015).

Negative-stain electron microscopy confirmed that the purified human complex (fractions E2–E3) had a Y-shaped structure and adopted multiple conformations (Fig. 1D, top; Fig. S2C, left), in agreement with recent cryo-electron microscopy structures (Gabel et al., 2022; Travis et al., 2023; Zupa et al., 2022). In our purification, the complete complex could be separated from partly assembled subcomplexes that eluted later from the size exclusion column (fractions E5–E7) (Fig. 1D, bottom; Fig. S2C, right). Together, these data indicate that the recombinant human augmin complex (hereafter denoted GFP-augmin) consisting of all full-length subunits is correctly assembled.

### Binding of augmin to microtubules *in vitro*

**Fig. 2.**
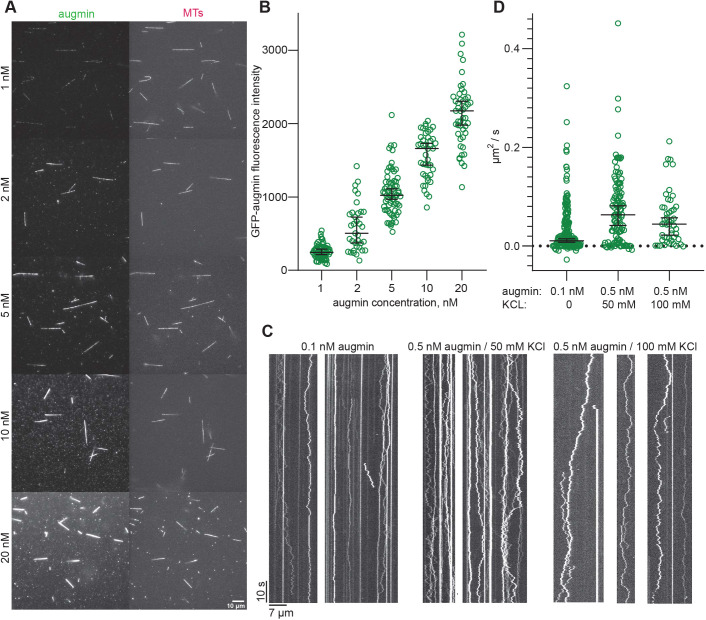
**Binding of the human augmin complex to microtubules *in vitro*.** (A) TIRF microscopy images showing binding of GFP-augmin at the indicated concentrations (from 1 nM to 20 nM) to TAMRA-microtubules (MTs). (B) Background-corrected GFP-augmin fluorescence intensity bound to stabilized microtubules at the indicated augmin concentrations (*n*=76, 35, 67, 42, 48). Black bars represent median values with 95% c.i. (C) TIRF microscopy kymographs showing the diffusion of single GFP-augmin molecules on microtubules. Concentrations and scale bars as indicated. (D) Diffusion coefficients of individual tracked augmin particles with and without added KCl (*n*=216, 113, 49). The diffusion coefficient was significantly increased in the experiments with added KCl at 50 mM (*P*<0.01; Mann–Whitney *U*-test) or 100 mM (*P*<0.01; Mann–Whitney *U*-test). Black bars represent median values with 95% c.i: 0.011 (0.006–0.015), 0.06 (0.04–0.08), 0.04 (0.02–0.05). Data are from three independent measurements (technical replicates) performed with one batch of purified augmin complex.

Total internal reflection fluorescence (TIRF) microscopy experiments demonstrated that the purified GFP-augmin complex at concentrations as low as 1 nM bound all along surface-immobilized GMPCPP and Taxol-stabilized TAMRA-labelled microtubules (Fig. 2A, top). Increasing the GFP-augmin concentration within the range of 1 nM to 20 nM increased the uniform binding to microtubules in a dose-dependent manner (Fig. 2A,B). At higher concentrations, the complex became insoluble, preventing us from reaching saturation of binding. A similar binding efficiency was observed recently for an augmin complex retrieved from cultured human cells (Zhang et al., 2022), whereas truncated *Xenopus* and human augmin complexes studied previously appeared to bind less efficiently, requiring higher complex concentrations to be used (Alfaro-Aco et al., 2020; Hsia et al., 2014).

Next, we examined the mode of GFP-augmin binding to microtubules using single-molecule TIRF microscopy imaging at lower augmin concentrations (Fig. 2C). Kymographs (time-space plots) revealed that some complexes bound statically, whereas the majority of the complexes bound in a diffusive manner to the microtubule lattice, mostly for the entire duration of the ∼2 min observation period. Increasing the KCl concentration to 50 mM or 100 mM reduced the number of observed events (Fig. S3A), which we compensated for by increasing the augmin concentration (Fig. 2C, right). We tracked the augmin molecules bound to microtubules with FIESTA (Ruhnow et al., 2011), calculated the diffusion coefficient for each track (Fig. 2D), and found that the diffusion coefficient was significantly increased in the experiments with added KCl (Fig. 2D). A more detailed fluorescence intensity analysis of the diffusing augmin particles revealed the presence of a mixture of single molecules and small oligomers of up to approximately ten molecules; larger oligomers tended to diffuse more slowly, probably as a result of increased friction (Fig. S3B).

To test whether the negatively charged C-terminal tubulin tails on the microtubule surface are required for the binding of the augmin complex, we removed the tails by subtilisin digestion (Fig. S3C). Untreated or subtilisin-treated microtubules were then immobilized on coverslips and the binding of 2 nM GFP-augmin complex was assayed by TIRF microscopy. Although augmin bound to native, non-treated microtubules, no binding was detected to subtilisin-treated microtubules without C-terminal tails (Fig. S3D). Collectively, these findings show that augmin complex–microtubule interactions are mediated by electrostatic interactions between the negatively charged microtubule tails and the positively charged unstructured N-terminal part of the HAUS8 subunit that represents the major microtubule-binding region of the complex (Wu et al., 2008).

### Importins bind to augmin and inhibit its microtubule binding

**Fig. 3.**
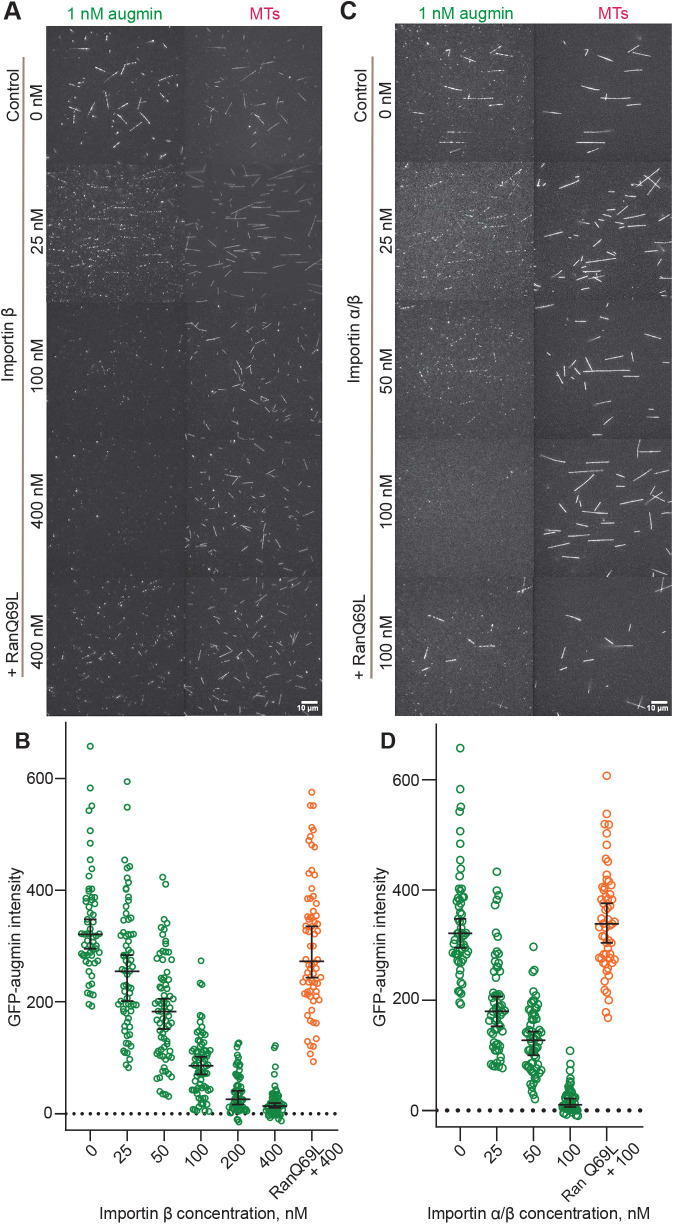
**Reconstitution of the regulation of augmin binding to microtubules by importins and Ran-GTP.** (A) TIRF microscopy images showing reduced binding of GFP-augmin (1 nM) to TAMRA-microtubules (MTs) in the presence of increasing concentrations of importin β (25–400 nM) and the rescue while adding constitutively active RanQ69L. (B) Background-corrected fluorescence intensities of GFP-augmin measured on microtubules in the presence of different importin β concentrations, as indicated, and with 3.5 µM RanQ69L added to the highest importin β concentration tested (conditions as in A and Fig. S4D) (*n*=59, 71, 73, 72, 62, 58, 70). Inhibition of binding by 400 nM importin β is almost completely reverted by adding 3.5 µM constitutively active RanQ69L (median, 272.9; 95 % c.i., 243.6–335.0), a slight but significant reduction compared with control without importin β (median, 321.2; 95 % c.i., 295.1–347.9), *P*=0.03 (Mann–Whitney *U*-test). (C) TIRF microscopy images showing reduced binding of GFP-augmin (1 nM) to MTs in the simultaneous presence of importin α and importin β (25–100 nM each) and the rescue while adding constitutively active RanQ69L. (D) Background-corrected fluorescence intensity of GFP-augmin measured on microtubules in the presence of different importin α/β concentrations as indicated, and with RanQ69L added to the highest importin α/β concentration tested (*n*=59, 60, 62, 57, 57). Inhibition of binding by 100 nM importin α/β is reverted by adding 3.5 µM RanQ69L (median, 338.4; 95% c.i., 303.8–375.8) compared with the control without importin α/β (median, 321.2; 95% c.i., 295.1–347.9), *P*=0.60 (Mann–Whitney *U*-test). Black bars represent the median values with 95% c.i. Data are from three independent measurements (technical replicates) performed with one batch of purified augmin complex.

During optimization of the human augmin purification, we noted the presence of insect cell importins in the mass spectrometry analysis, suggesting that these proteins might be augmin-binding partners (Fig. S4A). We, therefore, tested whether purified human importin α (KPNA2 subunit) and importin β (KPNB1 subunit) – referred to collectively as importin α/β – and a constitutively active form of human Ran (RanQ69L; Fig. S4B) can control the binding of augmin to microtubules. The addition of importin α to a TIRF microscopy-based binding assay had no effect on microtubule binding of augmin (Fig. S4C), in agreement with importin α being self-inhibited (Lott and Cingolani, 2011). However, the addition of importin β inhibited microtubule binding of augmin in a dose-dependent manner (Fig. 3A,B, green data; Fig. S4D). A mixture of both importin α and importin β inhibited augmin binding to microtubules even more efficiently, reaching almost complete inhibition at 100 nM importin α/β (Fig. 3C,D, green data). Adding RanQ69L at a concentration of 3.5 μM to the highest tested concentrations of importin β almost completely restored augmin binding to microtubules. For the mixture of both importins, addition of RanQ69L fully restored augmin binding to microtubules, demonstrating that Ran-GTP can relieve the inhibitory effect of the importins (Fig. 3B,D, orange data).

To quantify the strength of the interaction between the augmin complex and importin α/β, we used microscale thermophoresis and measured a dissociation constant of 13.1±3.65 nM (mean±s.d.), indicating strong binding (Fig. S5A).

These data show that the augmin complex is a direct target of the Ran-GTP pathway.

### Identification of a microtubule- and importin-binding site in HAUS8

**Fig. 4.**
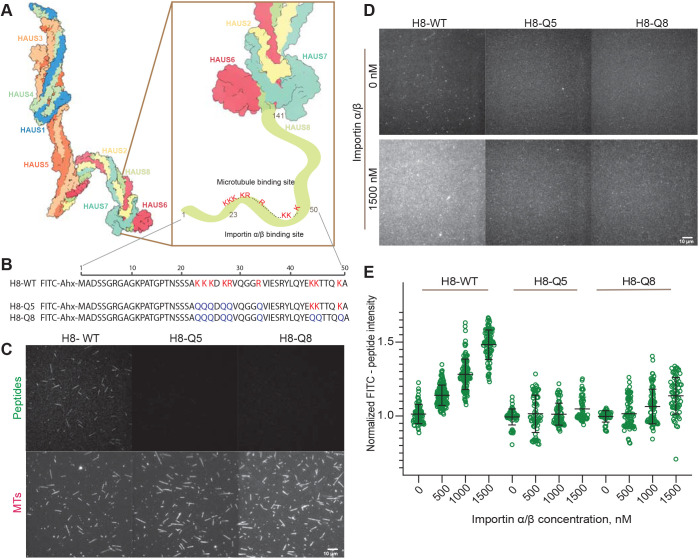
**Binding of the human augmin complex to importins and identification of the importin-regulated microtubule binding site of HAUS8.** (A) Schematic of the augmin complex (PDB 7SQK) and the importin-regulated microtubule binding site of the HAUS8 subunit. (B) Sequences of synthetic peptides derived from the N terminus of HAUS8 (amino acids 1–50). Positively charged amino acids corresponding to potential binding sites are marked in red. Amino acids mutated to glutamines are marked in blue. Ahx, 6-aminohexanoic acid. (C) TIRF microscopy images showing binding of FITC-labelled wild-type peptide (100 nM) and no binding of mutated FITC-peptides (100 nM) to TAMRA-microtubules (MTs). (D) TIRF microscopy images showing binding of 50 nM FITC-labelled wild-type peptide H8-WT, and mutated peptides H8-Q5 and H8-Q8 to the control channel (without importins) and to importin α/β that was immobilized on a glass surface (1500 nM). (E) Background-corrected normalized fluorescence intensities of FITC-peptides (50 nM) binding to surface-immobilized importin α/β. Importin concentrations used for immobilization were as indicated. The peptide H8-WT showed binding in a dose-dependent manner (slope: median, 0.48; 95% c.i. 0.27–0.68; calculated by linear regression with bootstrapping), whereas mutated peptides H8-Q5 and H8-Q8 did not bind importins (H8-Q5 slope: median, 0.00; 95% c.i., −0.10–0.20; H8-Q8 slope: median, 0.02; 95% c.i. −0.02–0.054). Green data represent intensities measured in different fields of view (*n*=79, 154, 120, 92, 68, 72, 74, 61, 49, 78, 84, 67); black bars represent median values with 95% c.i. Data in C–E are from three independent measurements (technical replicates) performed with one batch of purified augmin complex.

To identify the inhibitory importin binding site in the augmin complex, we inspected the N-terminal unstructured part of HAUS8 that is known to contain the major microtubule-binding site, although its exact location is unknown (Wu et al., 2008). Using the NLStradamus method for NLS motif prediction (Nguyen Ba et al., 2009), we identified a potential monopartite nuclear NLS motif (KKKDKR, amino acids 23–28 of HAUS8) that could have the ability to bind microtubules and importins in a competitive manner (Fig. 4A).

To test this hypothesis, we studied fluorescently labelled synthetic peptides that correspond to the first 50 amino acids of HAUS8 (Fig. 4B). Fluorescence microscopy showed that the peptide with the wild-type sequence (H8-WT) bound along the lattice of stabilized microtubules (Fig. 4C, left), whereas a mutant in which the five basic amino acids of the putative monopartite NLS were changed to glutamines (H8-Q5) did not bind microtubules (Fig. 4B,C, middle). Similarly, a peptide with three additional mutations in a second stretch of positively charged amino acids (H8-Q8) also did not bind microtubules (Fig. 4B,C, right). Compared with experiments with the complete augmin complex, we used here higher concentrations of wild-type peptide for comparable binding to microtubules. These results indicate that the identified potential NLS motif forms at least part of the microtubule-binding region of HAUS8, narrowing down the part of the N-terminal extension of HAUS8 that binds to microtubules.

To investigate whether the wild-type peptide binds importins, we immobilized importin α/β on a glass surface and added increasing concentrations of GFP-augmin (control) or fluorescent HAUS8 peptides (H8-WT, H8-Q5, H8-Q8), and imaged their binding by TIRF microscopy. GFP-augmin, as a positive control, showed dose-dependent binding to the importin surface (Fig. S5B,C). The wild-type peptide showed similar binding to the immobilized importins (Fig. 4D,E) in a dose-dependent manner. In contrast, the mutated peptides did not bind importins noticeably. These data demonstrate that the binding of importins to the identified monopartite NLS is specific. Together, these data suggest that HAUS8 binding to importins and to microtubules is competitive, revealing at least part of the mechanism by which importins and Ran-GTP control the binding of the augmin complex to microtubules.

## DISCUSSION

**Fig. 5.**
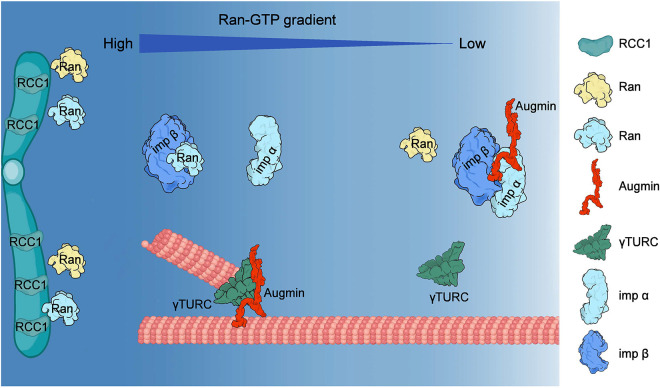
**Schematic of the Ran-GTP-dependent regulation of human augmin.** A Ran-GTP gradient forms around chromosomes on which the guanine-nucleotide-exchange factor RCC1 is enriched. High concentrations of Ran-GTP promote the dissociation of the NLS-containing augmin from inhibitory importin (Imp) α/β. The released augmin can bind to microtubules and trigger branched microtubule nucleation locally in the vicinity of chromosomes.

We show here that the human augmin complex is a direct target of Ran-GTP-mediated regulation. This regulation happens at the level of microtubule binding, which in turn will affect the recruitment of γTuRC and possibly other nucleation stimulating factors needed to trigger chromatin-mediated branched microtubule nucleation for correct spindle assembly during cell division (Fig. 5).

This finding provides a possible explanation for the observation that in some organisms, such as *Drosophila*, branched nucleation is dependent on the Ran-GTP pathway (Chen et al., 2015), but independent of TPX2 (Verma and Maresca, 2019), although TPX2 is an important SAF in *Xenopus laevis* eggs with well-established microtubule nucleation-stimulating activity (Petry et al., 2013). Our results also align with a recent *in vitro* reconstitution of branched microtubule nucleation using human proteins that did not include TPX2 (Zhang et al., 2022), indicating that TPX2 is not strictly required for branched microtubule nucleation in humans. Mechanistic differences may exist in different species.

We identified a previously undetected monopartite NLS motif in the N-terminal unstructured region of the HAUS8 subunit (Fig. 4A). This positively charged region is also present in HAUS8 orthologues in other species (for example in *Xenopus*, *Drosophila* and *Arabidopsis*; Hotta et al., 2012). Our observation of this NLS motif being important for microtubule binding narrows down the microtubule-binding region in this part of HAUS8 (Wu et al., 2008). Recent cryo-electron microscopy studies proposed that, additionally, calponin homology domains identified in HAUS6 and HAUS7 might also contribute to microtubule binding of the augmin complex (Gabel et al., 2022). Specificity of binding might be conferred by the folded calponin homology domains, and the affinity of the interaction may be controlled by the unstructured, positively charged part of HAUS8.

In agreement with our findings, a recent study identified two NLS motifs in the HAUS8 subunit of *Xenopus laevis* augmin, one of which does not exist in human HAUS8 (Kraus et al., 2023). These motifs in the *Xenopus* protein were also shown to be responsible, at least in part, for importin/Ran-GTP-dependent regulation of the binding of *Xenopus* augmin to microtubules. Therefore, the basic principle of direct importin/Ran-GTP regulation of augmin appears to be conserved between human and *Xenopus*, even if details of the NLS motifs may differ; this could explain why, in contrast to the human augmin, microtubule binding of *Xenopus* augmin could not be completely blocked by importins (Kraus et al., 2023).

Positively charged stretches that mediate or strengthen microtubule binding have been found in several microtubule-associated proteins, such as Tau (Lee et al., 1989), ASAP (also known as MAP9; Saffin et al., 2005) and NuSAP (also known as NUSAP1; Raemaekers et al., 2003). They are often a target of regulation, either by inhibitory importin binding [NuSAP (Ribbeck et al., 2006), HURP (also known as DLGAP5; Koffa et al., 2006), TPX2 (Safari et al., 2021)] or by inhibitory phosphorylation by kinases [doublecortin (Schaar et al., 2004), MAP6 (Baratier et al., 2006), Tau (Gong and Iqbal, 2008)] or by other post-translational modifications (HDAC6; Liu et al., 2012).

It has been reported that the N-terminal extension of human HAUS8 is also regulated by inhibitory phosphorylation by aurora A kinase (Tsai et al., 2011). Phosphorylation of amino acids that are closely located to the NLS motif identified here reduced microtubule nucleation in spindles of cultured human cells and impaired mitotic progression. Moreover, augmin with phosphorylated HAUS8 was enriched at the spindle poles (Tsai et al., 2011). This may suggest differential regulation of augmin in different parts of the spindle controlled by the combined action of importins and aurora A kinase.

Mass-spectrometry analysis of our purified recombinant augmin complex indicated that, despite strong phosphorylation of HAUS8, its N terminus was unphosphorylated, in agreement with the observed strong augmin binding to microtubules in the absence of importins. Compared with the entire augmin complex, the HAUS8-derived peptide H8-WT (amino acids 1–50) bound more weakly to microtubules. This is similar to the previous observation that the entire unstructured part of HAUS8 alone bound ten times more weakly to microtubules than an augmin subcomplex also containing the calponin homology domain comprising subunits HAUS6 and HAUS7 (T-II subcomplex) (Hsia et al., 2014), further supporting the notion of several parts of the complex contributing to microtubule binding.

In agreement with previous *in vitro* reconstitutions (Hsia et al., 2014; Zhang et al., 2022), we observed diffusive microtubule binding of the human augmin complex. Diffusion slowed down for larger augmin oligomers. The majority of the augmin particles were bound to microtubules for at least 2 min, compared with the measured dwell time of augmin of ∼1 min at nucleation sites in *Drosophila* cells (Verma and Maresca, 2019). Oligomerization and strong microtubule binding of augmin may be functionally important, given that more strongly bound augmin complexes observed in *Drosophila* cells were part of more active nucleation branch points (Verma and Maresca, 2019). Moreover, a recent *in vitro* reconstitution of branched microtubule nucleation with human proteins also revealed augmin oligomerization at active branch points (three or four augmin complexes per oligomer) (Zhang et al., 2022). Given the flexible structure of individual augmin complexes, multiple complexes may have to come together to ensure the preferential geometrical orientation of augmin- and γ-TURC-nucleated microtubules with the characteristic shallow angles observed for branched microtubule nucleation.

In conclusion, the augmin complex is a direct target of Ran-GTP-dependent regulation, which controls its binding to microtubules via importins. This sheds new light on the functioning of one of the key regulators of microtubule nucleation during cell division. Ran-GTP-dependent regulation of augmin can therefore be expected to control indirectly the recruitment of γTuRC by augmin for branched microtubule nucleation. Other SAFs, such as TPX2 in *Xenopus* or HURP in *Drosophila*, may have additional roles in regulating branched microtubule nucleation by either further promoting γTuRC recruitment or its activation by other mechanisms. This will be an interesting area for further future investigation.

## MATERIALS AND METHODS

### DNA constructs

The genes of the subunits of the human augmin complex [HAUS1 (Q96CS2; NM_138443.4); HAUS2 (Q9NVX0; NM_018097.3); HAUS3 (Q68CZ6; NM_001303143.2); HAUS4 (Q9H6D7; NM_001166269.2); HAUS5 (O94927; NM_015302.2); HAUS6 (Q7Z4H7; NM_017645.5); HAUS7 (Q99871; NM_001385482.1); HAUS8 (Q9BT25; NM_033417.2)] were synthesized in codon-optimized form for expression in *Trichoplusia ni* cells, including a polyhedrin promoter and terminator overhangs of the pLIB vectors for the assembly in a baculovirus biGBac expression vector (Weissmann and Peters, 2018; Weissmann et al., 2016).

The *HAUS1* with a C-terminal 3C-His_6_-tag and *HAUS3* were cloned into pBIG1a; the *HAUS2* with a C-terminal mEGFP-TEV-TwinStrep tag, *HAUS6* and *HAUS7* were cloned into pBIG1b; and *HAUS4*, *HAUS5* and *HAUS8* into pBIG1c. Eventually, all subunits of the augmin complex were joined into the pBIG2abc vector by Gibson assembly (Weissmann and Peters, 2018; Weissmann et al., 2016). The correct insertion of the genes was verified at each step of cloning using the restriction enzymes SwaI, PmeI and PacI. The correct sequence of the final vector was verified by Shotgun sequencing (MGH CCIB DNA Core, MA, USA).

The bacterial expression vectors for human importin α and β and RanQ69L, all with N-terminal His_6_-Ztag tags, have been described previously (Kronja et al., 2009; Roostalu et al., 2015).

### Protein expression and purification

#### Cell lines

Insect cells used for the baculovirus preparation were *Spodoptera frugiperda 21* (Sf21) (source EMBL), grown in Sf-900™ III SFM media (Gibco, 12658027), as published previously (Roostalu et al., 2018). Insect cells used for recombinant protein expression were *Trichoplusia ni* High Five insect cells, grown in Express Five™ SFM (Gibco, 10486025).

#### Augmin complex

The baculovirus preparation for recombinant human augmin complex expression was carried out according to the manufacturer's protocol (Bac-to-Bac system, Life Technologies) using *Escherichia coli* DH10EmBacY and Sf21 insect cells. Baculovirus-infected insect cells (Fig. S1D) were then frozen before cell lysis to generate stable viral stocks as described previously (Wasilko et al., 2009).

Human augmin complex expression was induced by adding 0.8 ml/l of frozen baculovirus-infected insect cells to a High Five insect cell culture grown to densities of ∼1.2×10^6^ cells/ml. Cells were harvested 60 h post-infection by centrifugation (15 min, 1000 ***g***, 4°C). Cell pellets were then washed with ice-cold PBS, centrifuged again (15 min, 1000 ***g***, 4°C), frozen in liquid nitrogen, and stored at −80°C.

Pellets from 2 l of insect cell culture were resuspended in ice-cold lysis buffer (50 mM phosphate buffer pH 7.5, 400 mM NaCl, 4 mM MgCl_2_, 5% glycerol, 2 mM TCEP, 1 mM PMSF) supplemented with protease inhibitors (Roche), DNase I (10 µg ml/ml, Sigma-Aldrich), PhosSTOP phosphatase inhibitor cocktail tablet (Roche) using twice the volume of buffer compared with the cell pellet. Resuspended cells were lysed using an Avestin EmulsiFlex-C5 homogenizer (two rounds). The lysate was then clarified by centrifugation (30 min, 50,000 ***g***, 4°C), and filtered through the 1.2 μm pore filters. Avidin was added at 50 μg/ml to the lysate. The supernatant was passed through a 1 ml StrepTrap HP column (GE Healthcare), the column was washed with 20 column volumes of the lysis buffer and the protein was eluted with 3 mM desthiobiotin (Sigma-Aldrich, D1411).

The eluate was concentrated using an Amicon Ultra-4 concentrator (Merck Millipore, 10 kDa cut-off), and incubated overnight at 4°C with TEV and 3C proteases to remove the C-terminal StrepTagII and C-terminal His_6_-tag from the HAUS2 and HAUS1 subunits, respectively. The solution was then passed over a Superose 6 Increase 10/30 size-exclusion chromatography column (GE Healthcare Life Sciences) that was pre-equilibrated in 50 mM phosphate buffer pH 7.5, 250 mM NaCl, 4 mM MgCl_2_, 3% glycerol, 0.5 mM TCEP. The collected fractions were analysed using SDS-PAGE and SYPRO Ruby staining, and the augmin complex-containing fractions were pooled, concentrated, flash-frozen and stored in liquid nitrogen until further use. The protein concentration was calculated from the absorbance measured at 280 nm using a NanoDrop One Microvolume UV-Vis spectrophotometer (Thermo Fisher Scientific) and the extinction coefficient of the entire augmin complex.

#### Importins α and β

Importins were expressed and purified as described previously (Roostalu et al., 2015) using a Protino Ni-TED resin for affinity purification, followed by removal of the N-terminal His_6_-Ztag by TEV protease cleavage and size exclusion chromatography using a Superdex 200 16/60 column (GE Healthcare). The aliquoted purified protein was stored in liquid nitrogen.

#### RanQ69L

The constitutively active Ran mutant was expressed in BL21-pLysS (Novagen) *E. coli* cells. Transformed cells were grown to a density of OD_600_=0.5 and induced with 0.5 mM IPTG. After overnight expression at 18°C, the cells were harvested and snap-frozen. The cells were lysed using an Avestin Emulsiflex in Ran buffer (25 mM MOPS pH 7.2, 150 mM NaCl, 1 mM MgCl_2_, 1 mM 2-mercaptoethanol), and centrifuged (30 min, 250,000 ***g***). The supernatant was loaded on a CoCl_2_-charged 5 ml HP chelating column (GE Healthcare), eluted with Ran buffer supplemented with 300 mM imidazole, and dialysed into Ran buffer. The Z-tag was cleaved off using His-tagged TEV protease and the solution was passed through the HP chelating column to remove the protease and uncleaved RanQ69L; 10 mM EDTA and 1 mM GTP were added and the solution was incubated on ice to allow for the loading of RanQ69L with GTP. Afterward, MgCl_2_ was gradually added to 10 mM and the protein was dialysed overnight into Ran buffer. The GTP concentration was then adjusted to 1 mM and the protein was concentrated (Vivaspin). Finally, sucrose and DTT were added to final concentrations of 125 mM and 1 mM, respectively. RanQ69L was snap-frozen and stored in liquid nitrogen.

#### Tubulin

Porcine tubulin was purified from the pig brain as described previously (Castoldi and Popov, 2003; Hyman et al., 1991)**.** The final buffer was BRB80 (80 mM PIPES pH 6.8, 1 mM EGTA, 1 mM MgCl_2_). The final concentration was measured at 280 nm using a NanoDrop One Microvolume UV-Vis spectrophotometer.

#### Tubulin labelling

Tubulin was labelled with 5(6)-TAMRA succinimidyl ester (Invitrogen, C1171) for fluorescence microscopy assays according to published methods (Consolati et al., 2022).

#### Microtubule polymerization

TAMRA-labelled microtubules or unlabelled subtilisin-treated microtubules were grown in the presence of 1 mM GMPCPP (Jena Bioscience) as described previously (Ustinova et al., 2020). Pelleted microtubules were resuspended in BRB80 buffer supplemented with 10 μM Taxol (Merck, T7191) and stored at room temperature until further use.

#### Microtubule subtilisin treatment

Microtubules (25 μM) were incubated with 50, 100 and 200 μg/ml subtilisin A (Carlsberg, P5380) in BRB80 buffer supplemented with 10 μM Taxol at 30°C for 1 h. The reaction was quenched by the addition of 2 mM phenylmethylsuphonyl fluoride (Thermo Fisher Scientific). The sample was then centrifuged at 14,000 ***g*** for 30 min at room temperature, and pelleted microtubules were dissolved in BRB80 buffer (supplemented with 10 μM Taxol) and stored at room temperature. The removal of the C-terminal tails was verified by SDS-PAGE (Fig. S4A). Microtubules treated with subtilisin A (Fig. S3C) were used for microscopy (Fig. S3D).

### SDS-PAGE and western blotting

Protein samples were resolved by SDS-PAGE (NuPAGE, 10% polyacrylamide gel, Thermo Fisher Scientific) (180 V, 70 min). For SYPRO Ruby staining, the SYPRO^®^ Ruby gel stain (Thermo Fisher Scientific) was used following the manufacturer's protocol. The stained gel was imaged using Bio-Rad Molecular Imager Gel Doc XR+.

For immunoblotting, the gel was transferred to an iBlot PVDF membrane using the iBlot™2 Gel Transfer Device (Thermo Fisher Scientific) under standard conditions. The membrane was blocked with 5% skimmed milk dissolved in PBS. Anti-HAUS tubulin antibodies (HAUS1: HPA040652, Atlas Antibodies, 1:500; HAUS2: GTX118734, 1:500, GeneTex; HAUS3: NBP3-05007, Novus Biologicals, 1:1000; HAUS4: LS-C155274, LSBio, 1:1000; HAUS5: A305-827A-M, Thermo Fisher Scientific, 1:1000; HAUS6: GTX118732, GeneTex, 1:1000; HAUS7: A305-557A, Thermo Fisher Scientific, 1:500; HAUS8: LS-B10617-50, LSBio, 1:1000) and secondary polyclonal swine anti-rabbit antibody conjugated to HRP (P0399, Agilent Technologies, 1:3000) were used for the detection of HAUS subunits. The chemiluminescent signal was detected on an iBright™ FL1500 imager (Thermo Fisher Scientific).

### Negative-stain electron microscopy

Augmin complex (8 µl at 7 nM) was added onto a transmission electron microscopy grid and blotted after 1 min. Then, 8 μl of uranyl acetate (2%) was deposited on the grid for 1 min before draining it off with a Whatman filter paper. Sample examination was performed with a JEM-400 (JEOL) transmission electron microscope at 120 kV. The microscope was equipped with a Gatan Orius SC1000 CCD Camera charge-coupled device camera (Gatan Inc.).

### TIRF microscopy assay

Flow chambers were prepared as described previously (Braun et al., 2011; Skultetyova et al., 2017; Ustinova et al., 2020). Briefly, microscope chambers were built from silanized coverslips (Menzel 1.5H; Marienfeld High Precision 1.5H) prepared following a previously described protocol using HCl for activation (Hyman, 1991; Wedler et al., 2022). Parafilm was used to space the glass coverslips to form channels of ∼0.1 mm thickness, ∼3 mm width and 18 mm length. Double-stabilized (GMPCPP/Taxol) microtubules prepared as described previously (Braun et al., 2011; Skultetyova et al., 2017; Ustinova et al., 2020) were attached to the glass surface in each chamber by anti-β-tubulin antibodies (Sigma-Aldrich, T7816, 20 μg/ml in PBS). Subsequently, various concentrations of mGFP-augmin with or without importins and RanQ69L, at concentrations as indicated in the main text and figure legends, were added to the chamber in the binding buffer: BRB80 supplemented with 1 mM TCEP (AMRESCO), 0.02 mg/ml κ-casein (Sigma-Aldrich, C0406), 1 mM MgCl_2_ (Sigma-Aldrich, M670), 40 mM D-glucose (Sigma-Aldrich, G7528), 3 mM ATP (Merck, A2383), 2 mM GTP (Jena Bioscience, NU-1047), 250 μg/ml glucose oxidase (SERVA, 22778) and 60 μg/ml catalase (Merck, C-40). BRB40 buffer (40 mM PIPES pH 6.8, 1 mM EGTA, 1 mM MgCl_2_) supplemented with 1 mM TCEP (AMRESCO), 0.02 mg/ml κ-casein (Sigma-Aldrich, C0406), 20 mM KCl (Merck, P3911), 1 mM MgCl_2_ (Sigma-Aldrich, M670), 40 mM D-glucose (Sigma-Aldrich, G7528), 0.005% F-127 (Sigma-Aldrich, P2443), 250 μg/ml glucose oxidase (SERVA, 22778), and 60 μg/ml catalase (Merck, C-40) was used in the assay with the peptides interacting with immobilized microtubules or immobilized importins.

### TIRF microscope set-up

TIRF microscopy was performed using an automated Nikon Eclipse T*i-*E with Perfect Focus System, a 100× oil immersion TIRF objective (NA=1.49; CFI SR Apo; Nikon), and an Andor iXon 888 Ultra EMCCD camera (Andor Technology) controlled by MetaMorph software (Molecular Devices). The sample was excited using 360° TIRF illumination (iLas2; Gataca Systems). The following filter combinations were used: a 488 nm TIRF filter set (TRF49904; Chroma) with an additional ET525/50 (Chroma) bandpass filter, and a 561 nm TIRF filter set (TRF49909; Chroma) with additional ET607/70 (Chroma) bandpass filter. Visualization was performed by sequential dual-colour imaging (switching between 488 nm and 561 nm excitation lasers to detect the mGFP-augmin and TAMRA-labelled microtubules, respectively). The image acquisition rate for the observation of augmin single-molecule movements was 1 frame per 113 ms. The microscope chamber was kept at 30°C using OkoLab temperature control.

### Interference reflection microscopy

Interference reflection microscopy (IRM) was implemented in the Nikon iLas2-TIRF microscopy set-up based on a published design (Simmert et al., 2018). We replaced the collimation lenses and aperture iris (L1, L2, L3, AI in Simmert et al., figure 2) by a fiber-coupled LED at 780 nm (M780F2; Thorlabs) with a multi-mode fiber (M28L01; Thorlabs) and large-beam fiber collimator (C40SMA-B; Thorlabs). The collimator, a field iris (SM1D12; Thorlabs) and an *xy* translation mount (CXY1A; Thorlabs) were mounted in a Ø1″ tube and placed at the widefield illumination port of the iLas2 adapter (Cairn Research). The beam was aligned via the *xy* translation mount onto a 700 nm short-pass dichroic (FF700-SDi01-25x36; Semrock) in the iLas2 adapter to combine both IRM imaging at 780 nm and TIRF microscopy imaging with 405 nm, 488 nm, 561 nm and 638 nm lasers. The IRM and TIRF beams were ultimately focused in the backfocal plane by a lens in the iLas2 adapter. IRM images were captured using a 50-50 dichroic, the 100× objective, an additional 1.5× magnification lens, a 655 long-pass filter (ET655lp; Chroma) and the Andor iXon 888 camera (see the ‘TIRF microscope set-up’ section). For images of unlabelled microtubules, 100 frames (acquired at 1 frame per 113 ms) were averaged and subtracted by the median of the IRM background (median of 101 images, offset by 1 µm in both directions between each frame).

### TIRF microscopy image analysis

Experimental data were processed with Fiji (Rueden et al., 2017; Schindelin et al., 2012) and FIESTA (Ruhnow et al., 2011).

To measure overall fluorescence intensities of microtubule-bound mGFP-augmin, we followed a previously described procedure (Ustinova et al., 2020). Briefly, areas around microtubules were manually determined; the fluorescence intensity of all pixels in these areas were then measured in the mGFP-augmin channel. After background subtraction, the average fluorescence intensity per pixel was calculated for each microtubule area, corresponding to one experimental data point. The data were plotted using GraphPad Prism software. Median values and 95% confidence intervals were calculated for each experimental condition.

Overall fluorescence intensities of mGFP-augmin or FITC-peptides to surface-immobilized importins were measured as described above, but intensities were measured in the entire field of view.

For diffusion measurements, mGFP-augmin particles were tracked using FIESTA. All tracks were manually verified and projected on an averaged path using a custom-written MATLAB script. From these positions, the one-dimensional diffusion coefficient for each complex was estimated using a covariance-based estimator (Vestergaard et al., 2014). The intensity of each complex was estimated using the averaged integrated intensity of the Gaussian fit (using only the first 20 frames of each track). The arbitrary intensity count was converted to the number of fluorescent molecules by dividing the intensity counts by the average integrated intensity of single mGFP-augmin particles (estimated in a separate photobleaching experiment).

### Microscale thermophoresis

A twofold dilution series of importins α/β was prepared in BRB80, 1 mM TCEP, 0.02 mg/ml κ-casein, 3 mM ATP and 2 mM GTP. An equal amount of 10 nM mGFP-augmin in the same buffer was added to the importin α/β dilution series (final importin α/β concentrations were 4.3 μM–0.131 nM). The reactions were incubated for 5 min at room temperature. The Monolith NT.115 microscale thermophoresis instrument with premium coated capillaries (NanoTemper) was used to measure binding curves. The excitation power was set to 40% and the MST power was set to 60%. The results were processed with MO.Affinity Analysis software.

### Peptide synthesis

A 50-residue peptide sequence from the N-terminal part of HAUS8 and two mutated versions were synthesized: wild type: MADSSGRGAGKPATGPTNSSSAKKKDKRVQGGRVIESRYLQYEKKTTQKA; mutant 1: MADSSGRGAGKPATGPTNSSSAQQQDQQVQGGQVIESRYLQYEKKTTQKA; mutant 2: MADSSGRGAGKPATGPTNSSSAQQQDQQVQGGQVIESQYLQYEQQTTQQA.

The peptides were assembled in a Liberty Blue instrument running 0.1 mmol-scale solid phase synthesis Fmoc protocols on Rink amide ProTide resin. Side chain protections were NG-2,2,4,6,7-pentamethyldihydrobenzofuran-5-sulphonyl (Arg), t-butyl (Ser, Thr, Asp, Glu), t-butyloxycarbonyl (Lys, Trp) and trityl (Asn, Gln). Double couplings with fivefold molar amounts of Fmoc-amino acid, Oxyma and diisopropylcarbodiimide in DMF, with microwave activation at 90°C, were used at every cycle. Fluorescent labelling at the N terminus was carried out by manual coupling of a 5(6)-carboxyfluorescein unit. Further details on the synthesis, cleavage, purification and analytical documentation of similar peptides can be found in Carrera-Aubesart et al. (2022).

### Mass spectrometry

Gels bands were destained (100 mM NH_4_HCO_3_ in 40% acetonitrile), reduced (DTT), alkylated (iodoacetamide) and dehydrated for trypsin digestion (Promega, V5113). Obtained peptide mix was desalted with a MicroSpin C18 column (The Nest Group, Inc.) prior to LC-MS/MS analysis. Samples were analysed using an Orbitrap Fusion Lumos or Orbitrap Eclipse mass spectrometer (Thermo Fisher Scientific) coupled to an EASY-nLC 1200 [Thermo Fisher Scientific (Proxeon)]. Peptides were separated by reversed-phase chromatography using a C18-column (Thermo Fisher Scientific).

Digested bovine serum albumin (New England Biolabs, P8108S) was analysed between each sample to avoid sample carryover and to assure the stability of the instrument. QCloud (Chiva et al., 2018) was used to control instrument longitudinal performance during the project.

Acquired spectra were analysed using the Proteome Discoverer software suite (v1.4 or 2.5, Thermo Fisher Scientific) and the Mascot search engine (v2.6, Matrix Science; Perkins et al., 1999). The data were searched against a SwissProt_Human database plus *Trichoplusia ni* reference proteome (UP000322000) (35,205 entries) or SwissProt_Human database plus sf21 UniProt reference proteome (UP000240619) (20,821 entries) plus a list (Beer et al., 2017) of common contaminants and all the corresponding decoy entries. Oxidation of methionine and N-terminal protein acetylation were used as variable modifications whereas carbamidomethylation on cysteines was set as a fixed modification. The false discovery rate (FDR) in peptide identification was set to a maximum of 5%.

### Statistical analysis

Images were processed with Fiji. Data plotting and statistical analysis were performed using the GraphPad Prism software, using the Mann–Whitney *U*-test for all mentioned *P*-values. Experimental data points represent median values with 95% c.i.; the values represent three independent measurements (technical replicates) performed with one batch of purified augmin complex.

The slopes in Fig. 4E and Fig. S5C were determined using a bootstrapping procedure: randomly picking (with replacement) one of the three replicates, randomly picking a data point from each replicate for each concentration (again with replacement), and calculating the slope via linear regression. This procedure was repeated *n*=1000 times and the median of bootstrap distribution along with 95% c.i. was used to estimate the slope and its confidence interval.

### Visualization

For visualization of the augmin complex in Fig. 4A UCSF Chimera was used (Pettersen et al., 2004). The following protein structures from PDB were used for schematic visualization: Ran (3GJ0), importin α (6IW8), importin β (1QGK), γTURC (6V6S), augmin (7SQK).

## Supplementary Material

10.1242/joces.261096_sup1 Supplementary information
