# Supplementary material for: Microtubule binding of the human augmin complex is directly controlled by importins and Ran-GTP
Source: J Cell Sci. 2023 Jun 26;136(12):jcs261096. doi: 10.1242/jcs.261096 (PMC10323247; doi:10.1242/jcs.261096)
Supplement: Supplementary information [file joces-136-261096-s1.pdf]

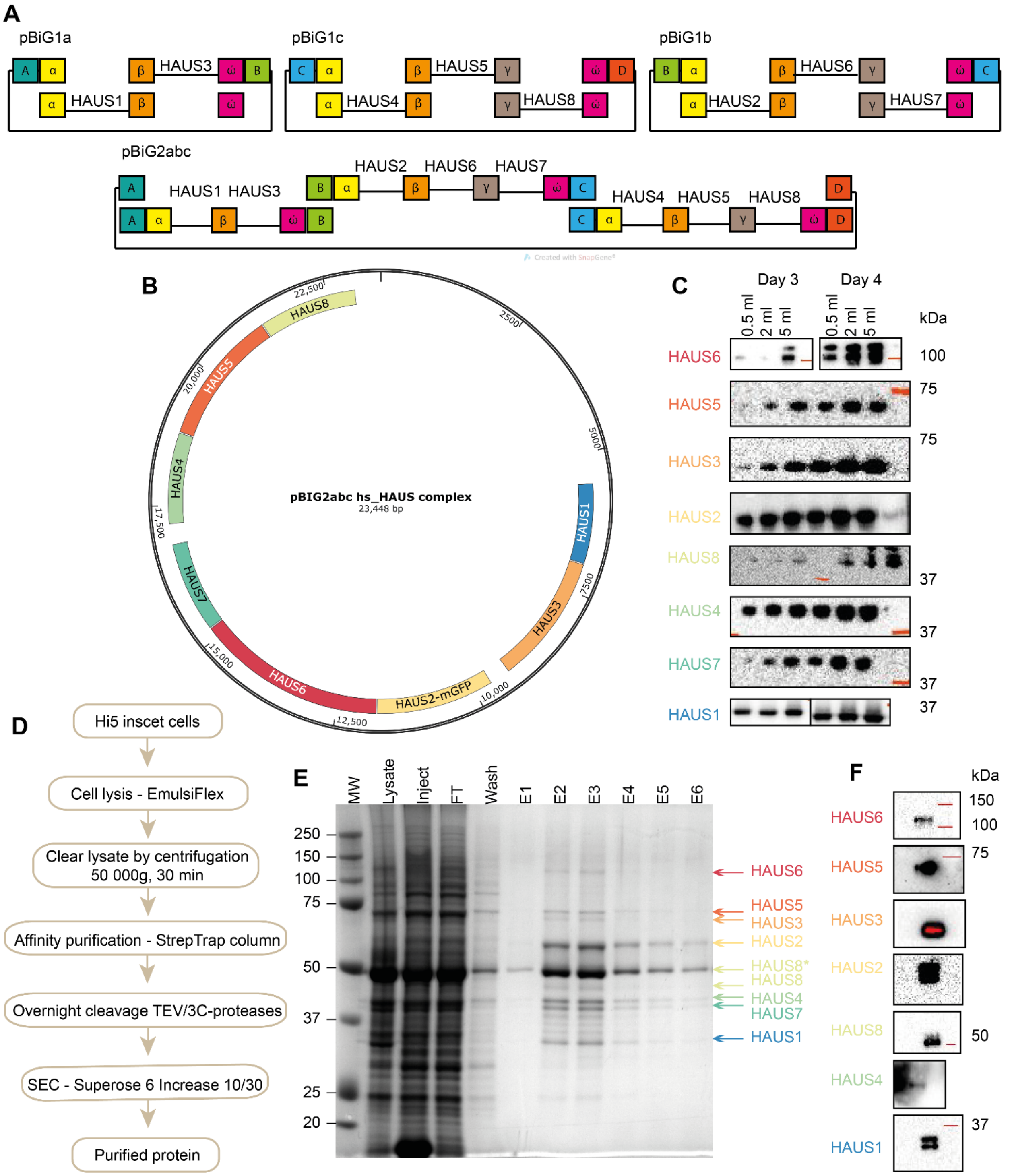

Fig. S1. Expression and purification of the human augmin complex. A. Schematic representation of the biGBac assembly vectors (pBIG1a/b/c, pBIG2abc) with the respective HAUS subunit genes. **B.** Schematic diagram of the assembled pBIG2abc vector with the 8 full-length

Journal of Cell Science • Supplementary information

HAUS subunits. **C.** Western blots of infected insect cells, demonstrating expression of all HAUS subunits. **D.** Flowchart of augmin complex purification. **E.** Coomassie-stained SDS-PAGE gel showing augmin complex fractions eluted from a StrepTrap HP column. HAUS8* indicates phosphorylated HAUS8. **F.** The presence of all HAUS subunits in the final protein sample was verified by western blotting.

Journal of Cell Science • Supplementary information

A

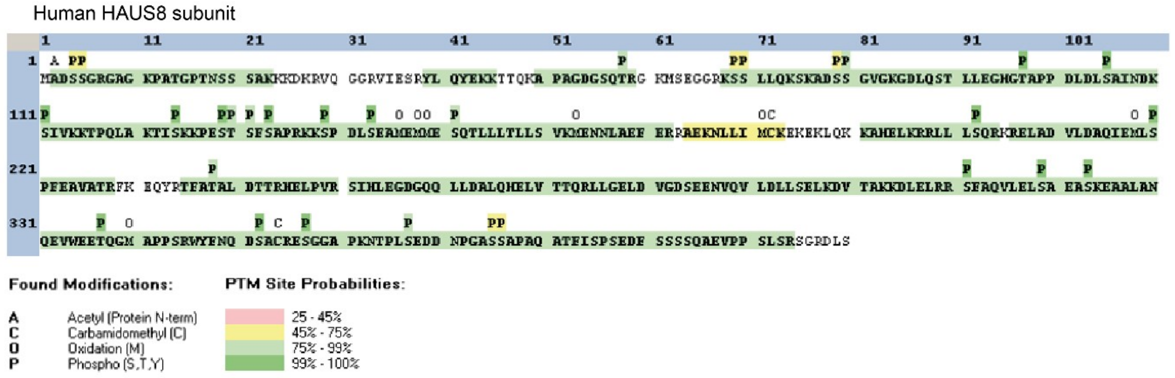

B

Phospho site table

S19-p	S131-p
S20-p	S133-p
S21-p	S139-p
Y39-p	S143-p
Y42-p	S151-p
S56-p	T153-p
S69-p	S160-p
S70-p	S220-p
S105-p	T235-p
T116-p	T238-p
T122-p	S311-p
S124-p	S352-p
S129-p	S357-p
T130-p	

C

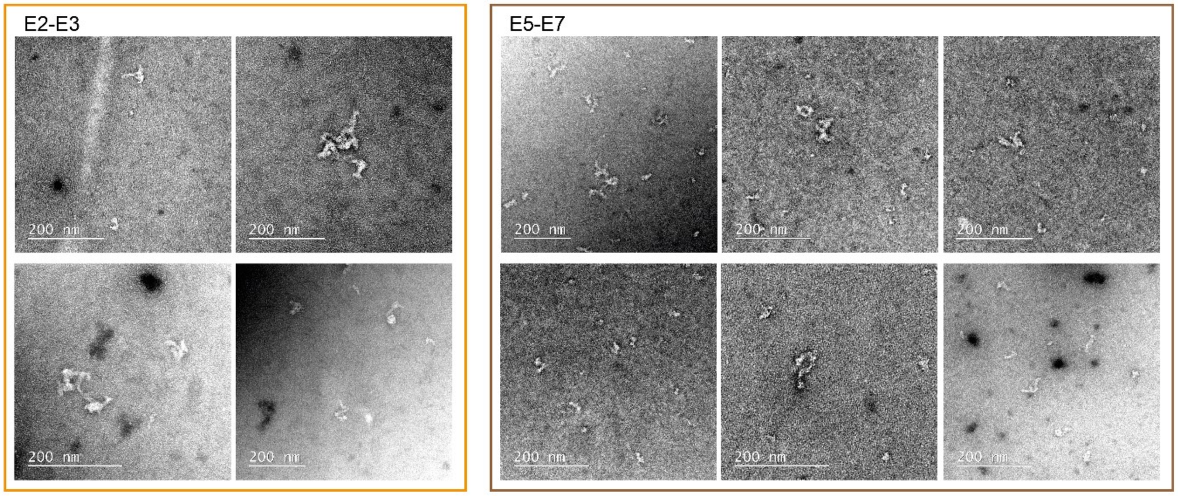

Fig. S2. HAUS8 phosphorylation state and recombinant human augmin complex structure. A. Posttranslational modifications of the HAUS8 sequence as detected by mass spectroscopy analysis, revealing a high degree of phosphorylation. **B.** Table of phosphorylation sites of HAUS8 reported in the literature (PhosphositePlus database) (Hornbeck et al. 2015). The

Journal of Cell Science • Supplementary information

phosphorylation sites identified by our mass spectroscopy analysis are marked green. **C.** Negative stain electron microscopy images of augmin complex, showing enrichment of complete complexes in fractions E2-E3 (left, orange box) and more partly assembled subcomplexes in fractions E5-E7 (right, brown box) eluting from the size exclusion chromatography column (see Fig. 1B-D).

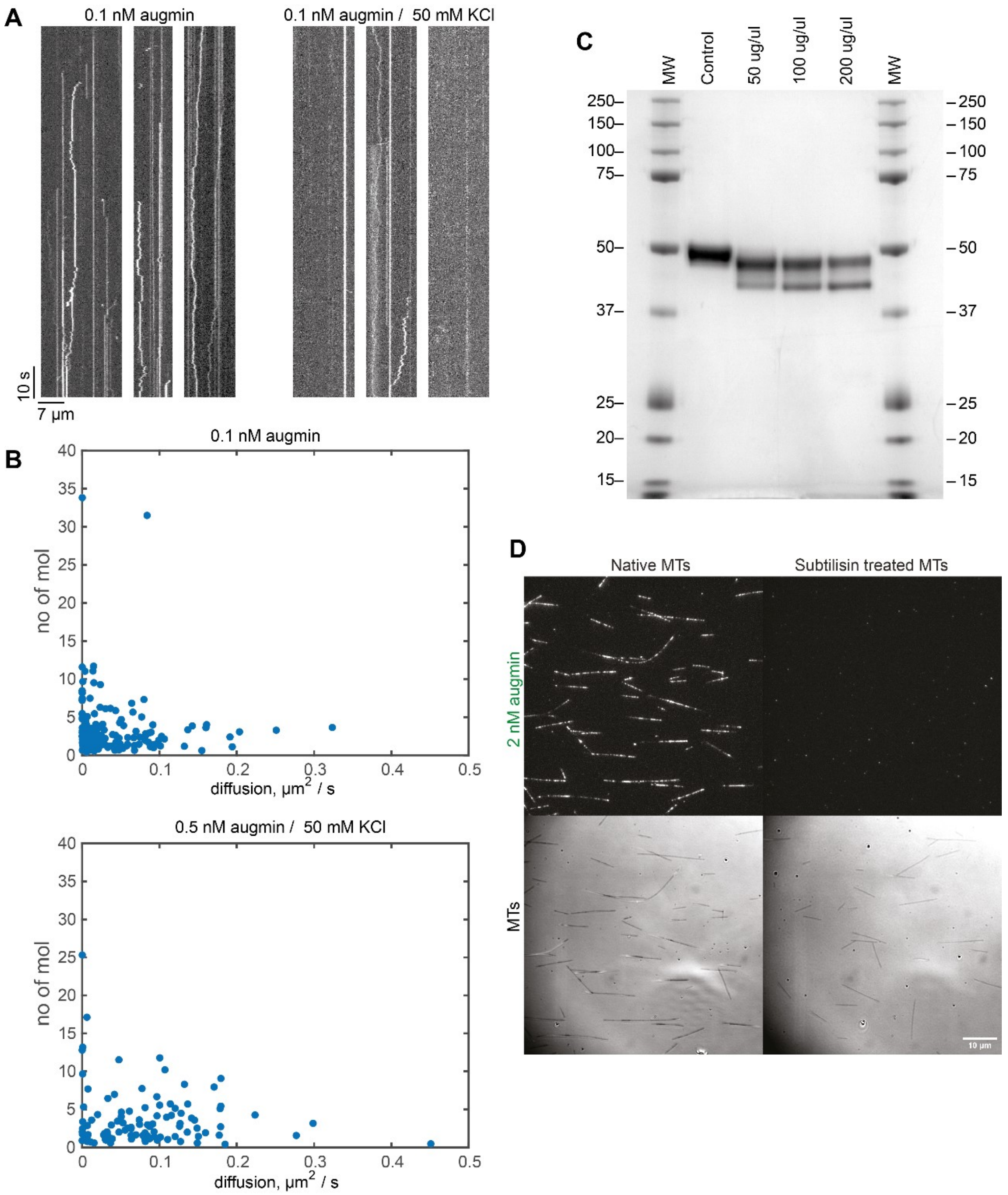

Fig. S3. Diffusion of GFP-augmin complexes on microtubules. A. TIRF microscopy kymographs showing the diffusion of single GFP-augmin particles on microtubules. In a higher ionic strength buffer fewer particles are observed. Concentrations and scale bars as indicated. **B.** Quantitative analysis of the diffusing GFP-augmin particles under the conditions shown in Fig. 2C. The diffusion coefficient of each tracked particle is plotted against the estimated number

Journal of Cell Science • Supplementary information

of augmin complexes per particle for the standard buffer (left) and the higher ionic strength buffer (right), showing that smaller particles and higher ionic strength promote faster diffusion. **C.** Coomassie-stained SDS-PAGE gel showing native and subtilisin-treated porcine microtubules. **D.** TIRF microscopy images showing binding of GFP-augmin to unlabeled microtubules: native and subtilisin treated. Microtubules were visualized using the interference reflection microscopy (IRM) set-up.

Journal of Cell Science • Supplementary information

A

Accession	Description	Coverage [%]	# Peptides
A0A7E5X1V3	importin-7 isoform X1 Trichoplusia ni	28	25
A0A7E5VE87	importin subunit alpha Trichoplusia ni	49	18
A0A7E5VDK5	importin-5 like Trichoplusia ni	42	36
A0A7E5VG59	importin subunit beta Trichoplusia ni	23	18
A0A7E5WB24	importin subunit alpha Trichoplusia ni	10	4
A0A7E5W216	importin subunit alpha Trichoplusia ni	14	5

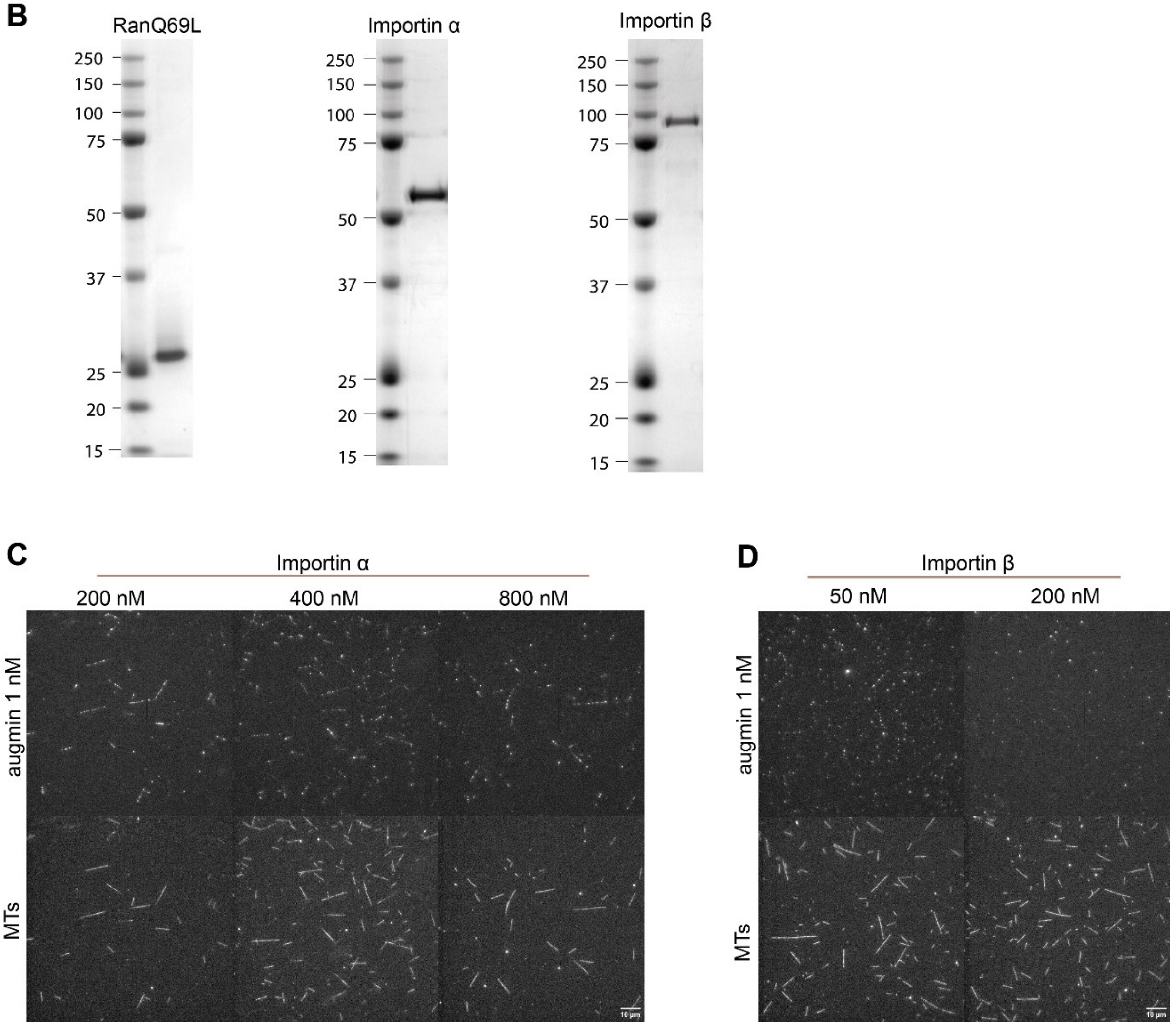

Fig. S4. Effect of importins on microtubule binding of the human augmin complex. A. Identification of several insect cell importins in a purification of the recombinant human augmin complex by mass spectroscopy analysis. **B.** Coomassie-stained SDS-PAGE gels showing purified RanQ69L, importin α and importin β. **C.** TIRF microscopy images showing binding of GFP-augmin (1 nM) in the presence of various concentrations of importin α, as indicated. The addition of importin α does not affect the binding of augmin to microtubules. **D.** TIRF microscopy images showing binding of GFP-augmin to TAMRA-microtubules (MTs) in the presence of 50 and 200 nM of importin β. Fluorescence intensities of bound GFP-augmin are quantified in Fig. 3B.

Journal of Cell Science • Supplementary information

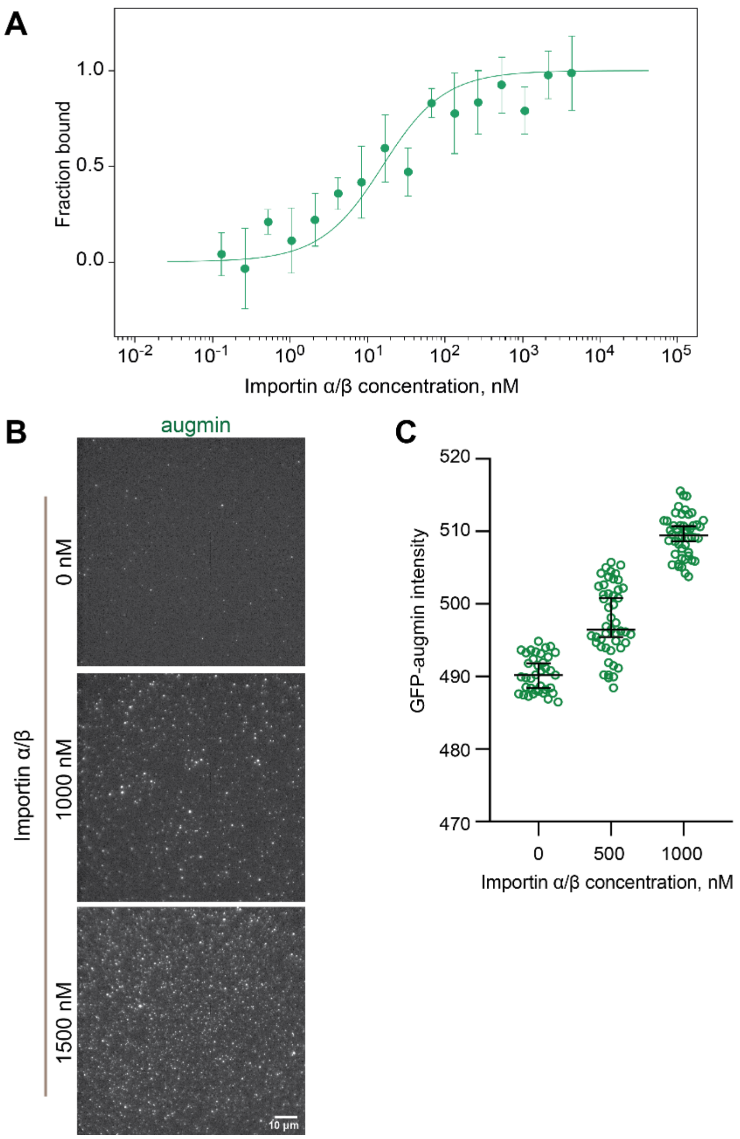

Fig. S5. Importin α/β bind specifically to the N-terminus of HAUS8. A. Quantification of concentration-dependent binding of augmin to importin α/β using microscale thermophoresis. GFP-augmin (5 nM) was mixed with different concentrations of importin α/β (4.3 µM – 0.131 nM). The obtained apparent dissociation constant was 13.1 ± 3.65 nM. Data points represent the mean ± S.D.; each data represents averaged number of 3 technical replicates (individual measurements). **B.** TIRF microscopy images showing binding of the GFP-augmin complex (2.5 nM) to importin α/β that was immobilized on a glass surface at two different importin concentrations as indicated. **C.** Fluorescence intensities of GFP-augmin complex (2.5 nM) binding to surface-immobilized importin α/β (slope 0.02 [0.01 0.03] median [95% CI], calculated by linear regression with bootstrapping). Importin concentrations used for immobilization were as indicated. Raw fluorescence intensities are shown. Green data points represent intensities measured in different fields of view, black bars represent median values with 95% CI (n = 37; 47; 49).

Journal of Cell Science • Supplementary information
