# Supplementary material for: Microtubule binding of the human augmin complex is directly controlled by importins and Ran-GTP
Source: J Cell Sci. 2023 Jun 26;136(12):jcs261096. doi: 10.1242/jcs.261096 (PMC10323247; doi:10.1242/jcs.261096)
Supplement: Supplementary file 1 [file joces-136-261096_review_history.pdf]

Microtubule binding of the human augmin complex is directly controlled by importins and Ran-GTP

Kseniya Ustinova, Felix Ruhnow, Maria Gili and Thomas Surrey
DOI: 10.1242/jcs.261096

Editor: Michael Way

Review timeline
Original submission: 19 February 2023
Editorial decision: 22 March 2023
First revision received: 28 April 2023
Accepted: 18 May 2023

Original submission

First decision letter

MS ID#: JOCES/2023/261096

MS TITLE: Microtubule binding of the human HAUS complex is directly controlled by importins and Ran-GTP

AUTHORS: Kseniya Ustinova, Felix Ruhnow, Maria Gili, and Thomas Surrey
ARTICLE TYPE: Research Article

We have now reached a decision on the above manuscript.

To see the reviewers' reports and a copy of this decision letter, please go to: https://submit-jcs.biologists.org and click on the 'Manuscripts with Decisions' queue in the Author Area. (Corresponding author only has access to reviews.)

As you will see, the reviewers gave very different reviews, with reviewer 1 raising a number of substantial criticisms and questions that prevent me from accepting the paper at this stage. They suggest, however, that a revised version might prove acceptable, if you can address their concerns. If you think that you can deal satisfactorily with the criticisms on revision, I would be pleased to see a revised manuscript.

Please ensure that you clearly highlight all changes text changes made in the revised manuscript in another colour. Please avoid using 'Tracked changes' in Word files as these are lost in PDF conversion.

I should be grateful if you would also provide a point-by-point response detailing how you have dealt with the points raised by the reviewers in the 'Response to Reviewers' box. Please attend to all of the reviewers' comments. If you do not agree with any of their criticisms or suggestions please explain clearly why this is so.

© 2023. Published by The Company of Biologists under the terms of the Creative Commons Attribution License (https://creativecommons.org/licenses/by/4.0/).

Reviewer 1

Advance summary and potential significance to field

Identifying the activation mode of the HAUS/augmin complex would provide new insights into spindle assembly and function.

Comments for the author

The manuscript by Ustinova et al presents new in vitro evidence that the HAUS/augmin complex, a central microtubule-dependent microtubule nucleator required for Ran-GTP-mediated amplification of microtubules during spindle assembly, is directly controlled by importin-binding to HAUS8. The authors do this by producing recombinant HAUS complex in insect, which certainly was not an easy feat, and then preform a variety of in vitro assays. While the overall finding is certainly of interest and in principle suitable for JCS, the current manuscript has several technical shortcomings that would need to be addressed prior to publication. Some lack of detail might be due to oversights in writing but in general, data seem preliminary and experimental results are overinterpreted. There is no statistical analysis. These concerns are laid out in detail below. If the manuscript is revised, it would also be very helpful to the reader to include a more informative diagram of the HAUS complex; how subunits interact, where are microtubule and importin binding sites?

Specific comments:
Fig. 1. I fully acknowledge that it is probably not easy to recombinantly make such a large and complicated protein complex in vitro, but I think some additional information for the purification would be relevant. Do the immunoblots in Fig. S1F correspond to the E2/3 fraction in Fig. 1C that is used for experiments? I think that should be explicitly stated in the figure legend and if this is the case I think Fig. S1F should be shown in the main figure.
Although immunoblotting in Fig. S1 seems to indicate that all subunits are present, the Coomassie gel shows that they are present in very different stoichiometries (i.e. very little HAUS6, lots of HAUS8). Is that expected? The authors should comment why this is the case and how it might affect activity of the HAUS complex. What do the asterisks indicate on the Coomassie gels in Figs. 1 and S1 (i.e. HAUS8*). Why does HAUS2 run as a doublet on the final gel filtration in Fig. 1, but not after the affinity column in Fig. S1? Also, gel filtration does not seem to improve purity. It is unclear why that was used
(Could one not have done an on-column TEV cleavage on the affinity matrix?).
Does E2/3 elute at the expected size from the gel filtration column? Is the affinity purification a StrepTrap or StrepTactin column? There seems to be some inconsistency in figures and text. Lastly, I don't think the statement that gel filtration separated full active complexes from subcomplexes is supported by the data. I can clearly see all protein bands also in E5-7. Although there is less qualitatively the bands seem to have a very similar stoichiometry. Similarly Fig. 1D does not support this statement either. There are small and big complexes in both conditions that are not clearly identifiable. There is no reference for the cryo-EM structure.
Fig. 2. How is concentration of the full active complex determined in these subsequent experiments because total protein concentration is clearly not the most informative measure here? Not sure that the absolute concentration values make a lot of sense given the substantial amounts of contaminants and the large excess of HAUS8 in the preparation. The long dwell time of HAUS on microtubules is surprising and unlike what is typically seen with other microtubule-binding proteins that associate and dissociate rapidly from microtubules. How is this 'processivity' achieved? Are there multiple microtubule binding sites or is this reflective of aggregated protein? The authors seem to think that there are oligomers (although it is completely unclear how that was quantified or if this is protein aggregation or a physiological oligomerization). Oligomers would diffuse slower possibly due to multiple microtubule binding sites. I don't think that has anything to do with friction. 50 mM KCl concentration seems low for these types of assays. Is there no salt in the standard reaction condition which does not seem to be included in the methods section. This is certainly not what these proteins would normally encounter in an intracellular environment.
Fig. 3. The effect of importin beta on HAUS microtubule-binding is possibly the most interesting result. However, what is the reason to use such a low HAUS concentration (1 nM) instead of a slightly higher one that gives more robust microtubule labelling such as 5 nM in Fig. 2. Why is a >100-fold excess of DA Ran Q69L needed to release HAUS from importin?

© 2023. Published by The Company of Biologists under the terms of the Creative Commons Attribution License (https://creativecommons.org/licenses/by/4.0/).

Fig. 4C. I don't understand the rationale why H8 peptides bind microtubules. Has the microtubule binding site of the HAUS complex not previously been described?
If the microtubule-binding site is truly in HAUS8, I think it would be required to do a similar experiment with full-length recombinant HAUS8 (with and without the mutations) and also compare microtubule binding to the whole complex. Given that HAUS8 is the most prominent band in the purified complex, this should not be a hard experiment to do. It would mean that the rest of the complex is irrelevant to this regulation. This seems important and should be tested.
Fig. 4E / S5. Binding of HAUS peptides to surface-immobilized importin seems like a very poor assay. There are lots of dots (aggregates?) with GFP-HAUS complex that looks very different from the peptides. It is not clear to me that this is not just background observed with the peptides. A different biochemical assay is a must here; fluorescence anisotropy, which should be simple to do with the reagents at hand or the thermophoresis shown in 4A.

Reviewer 2

Advance summary and potential significance to field

The HAUS/Augmin complex is an intriguing octameric protein assembly that mediates branched nucleation from existing microtubules. After a flurry of manuscripts from multiple spindle microtubule powerhouses, our understanding of the importance and architecture of the HAUS complex dramatically improved in 2022.

However, we need to learn more about the regulation of this crucial complex.

In the current manuscript, Ustinova and colleagues report the regulation of the HAUS complex by importins and Ran-GTP. In particular, the authors find that the intrinsically disordered N-terminus of HAUS-8 serves as both a nuclear localization signal/importin binding site and a known microtubule binding motif.

It was previously thought that the nucleation factor TPX2, which might stimulate branched microtubule nucleation, would be the target of importin. However, TPX2 seems to be optional for the process.

The novelty and quality of the work presented are outstanding.

The concentration-dependent effect of importin binding, physiological concentration range, and the 'rescue'

through constitutively active RanQ69L make this a convincing story.

Comments for the author

I would not object if the manuscript was published in its current form. The following questions and comments
should not be understood as a to-do list and distract from the timely publication of this manuscript:

Has anybody tried to test the effect of subtilisin digestion to remove the C-terminal tubulin tails on the
binding of HAUS8 or the entire complex?

Is the hypothesis that diffusive binding is a targeting mechanism that effectuates subsequently anchored
microtubule binding with the help of HAUS6?

The Kraus et al. bioRxiv# looks like it went through autocorrect and might need to be revised.

© 2023. Published by The Company of Biologists under the terms of the Creative Commons Attribution License (https://creativecommons.org/licenses/by/4.0/).

First revision

Author response to reviewers' comments

Point-by-point response to the reviewers' comments

Reviewer 1

Advance Summary and Potential Significance to Field:

Identifying the activation mode of the HAUS/augmin complex would provide new insights into spindle assembly and function.

Reviewer 1 Comments for the Author:

The manuscript by Ustinova et al presents new in vitro evidence that the HAUS/augmin complex, a central microtubule-dependent microtubule nucleator required for Ran-GTP-mediated amplification of microtubules during spindle assembly, is directly controlled by importin-binding to HAUS8. The authors do this by producing recombinant HAUS complex in insect, which certainly was not an easy feat, and then preform a variety of in vitro assays. While the overall finding is certainly of interest and in principle suitable for JCS, the current manuscript has several technical shortcomings that would need to be addressed prior to publication. Some lack of detail might be due to oversights in writing, but in general, data seem preliminary and experimental results are overinterpreted. There is no statistical analysis. These concerns are laid out in detail below. If the manuscript is revised, it would also be very helpful to the reader to include a more informative diagram of the HAUS complex; how subunits interact, where are microtubule and importin binding sites?

We thank the reviewer for the evaluation of our work. We have added a statistical analysis for the data presented in Figs. 2D, 3B, 3D, 4E, S5C (in the legends) and a scheme of the HAUS complex in Fig. 4A, as suggested (using the same HAUS subunits colors throughout the article for clarity).

Specific comments:

Fig. 1. I fully acknowledge that it is probably not easy to recombinantly make such a large and complicated protein complex in vitro, but I think some additional information for the purification would be relevant. Do the immunoblots in Fig. S1F correspond to the E2/3 fraction in Fig. 1C that is used for experiments? I think that should be explicitly stated in the figure legend and if this is the case I think Fig. S1F should be shown in the main figure.

The immunoblots in Fig. S1F indeed correspond to the E2-E3 fraction in Fig. 1C. It is now stated in our text (line 80-82) and in the legend of Fig. S1F.

Although immunoblotting in Fig. S1 seems to indicate that all subunits are present, the Coomassie gel shows that they are present in very different stoichiometries (i.e. very little HAUS6, lots of HAUS8). Is that expected?

We acknowledge that in an ideal world, we expect to see more stoichiometric gel band intensities, but often this can be difficult to achieve for larger complexes. For example, a previously published and cited purification of a *Xenopus laevis* HAUS complex that was expressed in insect cells shows a gel band pattern similar to the human HAUS complex that we have purified (Song et al., 2018). Moreover, our negative stain electron microscopy data demonstrate that the full complex with its typical Y shape is enriched in the gel filtration fractions E2-E3 that we use for our microtubule binding experiments (in contrast to the later fractions E5 to E7 that also contain substantial amounts of different subcomplexes and that we do not use in our characterizations of the properties of the HAUS complex).

The authors should comment why this is the case and how it might affect activity of the HAUS complex.

© 2023. Published by The Company of Biologists under the terms of the Creative Commons Attribution License (https://creativecommons.org/licenses/by/4.0/).

It is useful to note that the GFP tag that we observe in our fluorescence microscopy experiments is fused to the HAUS2 subunit which is not known to contribute to microtubule binding, whereas the N-terminal tail of HAUS8 is known to bind to microtubules (Hsia et al., 2014; Wu et al., 2008) and is controlled by importins, as we show here (in agreement with a very recently published study using *Xenopus* HAUS (Kraus et al., 2023)), supporting the notion of HAUS complex formation.

What do the asterisks indicate on the Coomassie gels in Figs. 1 and S1 (i.e. HAUS8*). Why does HAUS2 run as a doublet on the final gel filtration in Fig. 1, but not after the affinity column in Fig. S1?

HAUS2 runs as a double band in Fig. 1 as a consequence of incomplete TEV cleavage of the affinity tag fused to this subunit. The asterisk indicates the fraction of uncleaved HAUS2. We have clarified this in the revised legend of Fig. 1C and the main text (lines 78). As the reviewer notes, HAUS2 does not run as a double band directly after elution from the affinity column and before TEV cleavage (as expected).

The asterisk next to HAUS8 in Fig. S1E indicates the fraction of HAUS8 that migrates with an apparent molecular weight that is higher than expected (around 50 kDa instead of the expected, and also additionally observed 45 kDa band). This is due to the phosphorylation of this subunit as reported before (Tsai et al., 2011), and confirmed by our mass spectrometry data (Fig. S2A, lines 83-85). We have clarified the meaning of the asterisk in the revised legend of Fig. S1E.

Also, gel filtration does not seem to improve purity. It is unclear why that was used (Could one not have done an on-column TEV cleavage on the affinity matrix?).

We demonstrate by negative stain electron microscopy (Fig. 1D, S2C) that gel filtration is needed to enrich the complete HAUS complex, as described in our text, and similar to previous studies (Song et al., 2018; Zupa et al., 2022).

Does E2/3 elute at the expected size from the gel filtration column?

The E2-E3 fractions elute earlier from the gel filtration column than expected for a globular protein (but not in the void volume). This is expected given the elongated shape of the complex and has been observed before with the same gel filtration column (Hsia et al., 2014).

Is the affinity purification a StrepTrap or StrepTactin column? There seems to be some inconsistency in figures and text.

We thank the reviewer for spotting this inconsistency. We have used a StrepTrap HP column (GE Healthcare) and corrected this in the legend of Fig. S1E.

Lastly, I don't think the statement that gel filtration separated full active complexes from subcomplexes is supported by the data. I can clearly see all protein bands also in E5-7. Although there is less, qualitatively the bands seem to have a very similar stoichiometry. Similarly, Fig. 1D does not support this statement either. There are small and big complexes in both conditions that are not clearly identifiable. There is no reference for the cryo-EM structure.

As the reviewer states, all subunits are present in the fractions E5-7, but not all are assembled into the full complex, as our negative stain electron microscopy data show. One can clearly see more smaller particles that are not Y-shaped in fractions E5-E7 compared to E2-E3 (Fig 1D, Fig. S2C), also in agreement with the different elution volumes of these fractions. This result agrees with previously reported purifications of HAUS reporting the separation of full complexes from subcomplexes (Song et al., 2018; Zupa et al., 2022). We added the missing citations of the recent cryo-electron microscopy studies to the main text.

Fig. 2. How is concentration of the full active complex determined in these subsequent experiments because total protein concentration is clearly not the most informative measure here? Not sure that the absolute concentration values make a lot of sense given the substantial amounts of contaminants and the large excess of HAUS8 in the preparation.

© 2023. Published by The Company of Biologists under the terms of the Creative Commons Attribution License (https://creativecommons.org/licenses/by/4.0/).

As observed also by others (Hsia et al., 2014; Song et al., 2018), we are able to purify only relatively small amounts of the HAUS complex. Therefore, we used the sensitive SyproRuby-staining method to visualize the composition of gel filtration-purified HAUS from SDS-PAGE gels. Given the sensitivity of the SyproRuby staining method, minor contaminants are also visualized (that would be invisible in a Coomassie-stained gel). We used a NanoDrop One Microvolume UV-VIS spectrophotometer (Thermo Fisher Scientific) to measure the approximate concentration of the HAUS complex using the extinction coefficient of the entire HAUS complex, making the simplifying assumption that all subunits are present in stoichiometric amounts. This is described in the Methods. We acknowledge that we might overestimate to some extent the concentration of the complete HAUS complex, but it does not affect our conclusions given that the concentrations are calculated always in the same manner for all experiments.

The long dwell time of HAUS on microtubules is surprising and unlike what is typically seen with other microtubule-binding proteins that associate and dissociate rapidly from microtubules. How is this ‘processivity’ achieved? Are there multiple microtubule binding sites or is this reflective of aggregated protein?

This question is addressed in Fig. 2C and Fig. S3A, B where we have quantified the fluorescence intensities of the HAUS particles binding to microtubules. We show that HAUS particles stay indeed bound mostly for the entire duration of the ~ 2 min observation period and that the particles are heterogeneous ranging in size mostly from 1 to 10 HAUS complexes with the majority being around ~ 5 molecules. Hardly any much larger particles are observed, excluding major aggregate formation. The oligomerization of the complex may lead to increased affinity of the HAUS complex to microtubules, in agreement with a recent in vitro study of the human HAUS complex isolated from human cells where dwell times > 6 min were observed (Zhang et al., 2022). We acknowledge that the literature also provides examples of more short-lived in vitro binding events (Hsia et al., 2014), and future work will need to show whether purification or buffer differences (type of casein, presence of sucrose, taxol) may affect the behavior of the HAUS complex in vitro. Interestingly, in *Drosophila* cells, relatively long dwell times in the range of ~ 1 min were reported for augmin binding to microtubules (Verma and Maresca, 2019), potentially suggesting that oligomer formation is physiological and may slow down the binding/unbinding turnover. We cite these publications mentioned above.

The authors seem to think that there are oligomers (although it is completely unclear how that was quantified or if this is protein aggregation or a physiological oligomerization). Oligomers would diffuse slower possibly due to multiple microtubule binding sites. I don’t think that has anything to do with friction.

We describe in our Methods section how the number of HAUS complexes in the oligomers was calculated (in the section 'TIRF microscopy image analysis', last paragraph). mGFP-HAUS particles were tracked using the tracking software FIESTA. All tracks were manually verified and projected on an averaged path. From these positions, the one-dimensional diffusion coefficient for each complex was estimated using a covariance-based estimator (Vestergaard et al., 2014). The number of HAUS molecules in the particles was estimated by dividing the particle intensity by the average intensity of single mGFP-HAUS particles which was estimated in a separate photobleaching experiment.

50 mM KCl concentration seems low for these types of assays. Is there no salt in the standard reaction condition, which does not seem to be included in the methods section. This is certainly not what these proteins would normally encounter in an intracellular environment.

The buffer for our TIRF microscopy assays contains 80 mM PIPES at pH 6.8. This buffer has a relatively high ionic strength given that PIPES can carry up to two negative charges. In addition to the experiments with an additional 50 mM KCl (Fig. 2C, D, S3A, B), in the revised manuscript we have added also experiments with an additional 100 mM KCl, showing that also here HAUS complexes bind in a diffusive manner to the microtubule lattice and show further reduced binding, demonstrating that electrostatic interactions play an important role for HAUS binding to microtubules (new Fig. S3D).

© 2023. Published by The Company of Biologists under the terms of the Creative Commons Attribution License (https://creativecommons.org/licenses/by/4.0/).

Fig. 3. The effect of importin beta on HAUS microtubule-binding is possibly the most interesting result. However, what is the reason to use such a low HAUS concentration (1 nM) instead of a slightly higher one that gives more robust microtubule labelling such as 5 nM in Fig. 2. Why is a >100-fold excess of DA Ran Q69L needed to release HAUS from importin?

We have chosen a low HAUS concentration (1 nM) in order not to provoke excessive oligomer formation. HAUS binding can easily be visualized at this concentration. We then tested the effect of different concentrations of importins. Essentially complete inhibition of HAUS binding to microtubules was observed at 400 nM importin β and 100 nM importin α/β. We added then an 8.75-fold excess of Ran Q69L (3.5 μM) to the highest tested concentrations of importin β (400 nM) and for simplicity used the same Ran Q69L concentration for the rescue experiment in the presence of importin α/β. Previous studies used a 3-fold and 10-fold excess of Ran over importins (Kraus et al., 2023; Ribbeck et al., 2006), which is in a similar range as in our experiments.

Fig. 4C. I don't understand the rationale why H8 peptides bind microtubules. Has the microtubule binding site of the HAUS complex not previously been described? If the microtubule-binding site is truly in HAUS8, I think it would be required to do a similar experiment with full-length recombinant HAUS8 (with and without the mutations) and also compare microtubule binding to the whole complex. Given that HAUS8 is the most prominent band in the purified complex, this should not be a hard experiment to do. It would mean that the rest of the complex is irrelevant to this regulation. This seems important and should be tested.

Wu et al. 2008 indeed reported that the N-terminal part of HAUS8 (aa 1-141) binds to microtubules. Here we go beyond that finding by showing that this long N-terminal part of HAUS8 contains a loose NLS consensus sequence from amino acids 23 to 49 that is important for microtubule binding. In our Discussion (paragraph #4), we cite a recent publication that nicely complements and supports our findings (Kraus et al., 2023). Two NLS motifs were identified in the N-terminal part of *Xenopus laevis* HAUS8. These motifs were also shown to be responsible, at least in part, for importin/Ran-GTP-regulated binding of *Xenopus* augmin to microtubules. The recombinantly expressed N-terminal part of HAUS8 (aa 1-150) interacted with microtubules similar to the HAUS complex, while the mutations in the NLS sites lead to reduced binding to microtubules. These results confirm the importance of the N-terminal part of HAUS8 and the regulation of the HAUS complex by importins/Ran. Therefore, the basic principle of direct importin/Ran-GTP regulation of the HAUS complex appears to be conserved between human and *Xenopus*.

Fig. 4E / S5. Binding of HAUS peptides to surface-immobilized importin seems like a very poor assay. There are lots of dots (aggregates?) with GFP-HAUS complex that looks very different from the peptides. It is not clear to me that this is not just background observed with the peptides. A different biochemical assay is a must here; fluorescence anisotropy, which should be simple to do with the reagents at hand or the thermophoresis shown in 4A.

To improve the data quality, for the revised version of the manuscript we increased the peptide concentrations fivefold which resulted in an increased fluorescence signal of peptide bound to surface-immobilized importins. These new data are shown in Fig. 4D. Our previous conclusions continue to be supported by the improved-quality data.

We have tried to measure the binding affinity of peptides to importins using microscale thermophoresis but did not obtain a reliable signal indicating that the affinity is too weak for this method. The strength of the TIRF microscopy assay is its much higher sensitivity. The failure of the mutated peptides to bind to the importins demonstrates that the binding of the wildtype peptide is specific. We do not see a reason for concluding that this assay is not suitable. Moreover, our results align well with a very recently published study about the N-terminal part of HAUS 8 in the *Xenopus* HAUS complex using complementary methods (Kraus et al., 2023).

We also would like to clarify that in the previous version of the manuscript, the TIRF microscopy images of the importin binding assays were shown at higher magnification, which might have led to the impression that HAUS complex binding looked more 'dotty' than in the microtubule binding experiments. In the revised version we show the importin binding experiments in Fig. S5B at the same magnification as the microtubule binding experiments to avoid confusion.

© 2023. Published by The Company of Biologists under the terms of the Creative Commons Attribution License (https://creativecommons.org/licenses/by/4.0/).

Reviewer 2

Advance Summary and Potential Significance to Field:

The HAUS/Augmin complex is an intriguing octameric protein assembly that mediates branched nucleation from existing microtubules. After a flurry of manuscripts from multiple spindle microtubule powerhouses, our understanding of the importance and architecture of the HAUS complex dramatically improved in 2022.

However, we need to learn more about the regulation of this crucial complex.

In the current manuscript, Ustinova and colleagues report the regulation of the HAUS complex by importins and Ran-GTP. In particular, the authors find that the intrinsically disordered N-terminus of HAUS-8 serves as both a nuclear localization signal/importin binding site and a known microtubule binding motif.

It was previously thought that the nucleation factor TPX2, which might stimulate branched microtubule nucleation, would be the target of importin. However, TPX2 seems to be optional for the process.

The novelty and quality of the work presented are outstanding.

The concentration-dependent effect of importin binding, physiological concentration range, and the 'rescue' through constitutively active RanQ69L make this a convincing story.

We thank the reviewer for the positive evaluation of our work.

Reviewer 2 Comments for the Author:
I would not object if the manuscript was published in its current form. The following questions and comments should not be understood as a to-do list and distract from the timely publication of this manuscript:

Has anybody tried to test the effect of subtilisin digestion to remove the C-terminal tubulin tails on the binding of HAUS8 or the entire complex?

We thank the reviewer for this suggestion. We performed new experiments demonstrating that binding of the HAUS complex to subtilisin-treated microtubules is strongly reduced. This result provides additional evidence for electrostatic interactions between the N-terminus of HAUS8 and the C-terminal tails of tubulin being important for HAUS binding to microtubules. These data are shown in the new Fig. S3D.

Is the hypothesis that diffusive binding is a targeting mechanism that effectuates subsequently anchored microtubule binding with the help of HAUS6?

Recent cryo-EM structures of the HAUS complex suggest that the HAUS6 subunit may be involved in the binding of the complex to microtubules (Gabel et al., 2022). Therefore, several binding sites may indeed cooperate, as we mention in the Discussion, which is an interesting area for future investigations.

The Kraus et al. bioRxiv# looks like it went through autocorrect and might need to be revised.

We thank the reviewer for spotting this formatting mistake which is now corrected (referencing the revised version of this manuscript that has in the meantime been published).

References cited in this response letter:

Gabel, C. A., Li, Z., DeMarco, A. G., Zhang, Z., Yang, J., Hall, M. C., Barford, D. and Chang, L. (2022). Molecular architecture of the augmin complex. *Nat Commun* **13**, 5449.
Hsia, K. C., Wilson-Kubalek, E. M., Dottore, A., Hao, Q., Tsai, K. L., Forth, S., Shimamoto,

© 2023. Published by The Company of Biologists under the terms of the Creative Commons Attribution License (https://creativecommons.org/licenses/by/4.0/).

Y., Milligan, R. A. and Kapoor, T. M. (2014). Reconstitution of the augmin complex provides insights into its architecture and function. *Nat Cell Biol* **16**, 852-63.
Kraus, J., Travis, S. M., King, M. R. and Petry, S. (2023). Augmin is a Ran-regulated spindle assembly factor. *J Biol Chem*, 104736.
Ribbeck, K., Groen, A. C., Santarella, R., Bohnsack, M., Raemaekers, T., Köcher, T., Gentzel, M., Görlich, D., Wilm, M., Carmeliet, G. et al. (2006). NuSAP, a mitotic RanGTP target that stabilizes and cross- links microtubules. *Mol Biol Cell*.
Song, J. G., King, M. R., Zhang, R., Kadzik, R. S., Thawani, A. and Petry, S. (2018). Mechanism of how augmin directly targets the gamma-tubulin ring complex to microtubules. *J Cell Biol* **217**, 2417-2428.
Tsai, C. Y., Ngo, B., Tapadia, A., Hsu, P. H., Wu, G. and Lee, W. H. (2011). Aurora-A phosphorylates Augmin complex component Hice1 protein at an N-terminal serine/threonine cluster to modulate its microtubule binding activity during spindle assembly. *J Biol Chem* **286**, 30097-106.
Verma, V. and Maresca, T. J. (2019). Direct observation of branching MT nucleation in living animal cells. *J Cell Biol* **218**, 2829-2840.
Wu, G., Lin, Y. T., Wei, R., Chen, Y., Shan, Z. and Lee, W. H. (2008). Hice1, a novel microtubule- associated protein required for maintenance of spindle integrity and chromosomal stability in human cells. *Mol Cell Biol* **28**, 3652-62.
Zhang, Y., Hong, X., Hua, S. and Jiang, K. (2022). Reconstitution and mechanistic dissection of the human microtubule branching machinery. *J Cell Biol* **221**.
Zupa, E., Wurtz, M., Neuner, A., Hoffmann, T., Rettel, M., Bohler, A., Vermeulen, B. J. A., Eustermann, S., Schiebel, E. and Pfeffer, S. (2022). The augmin complex architecture reveals structural insights into microtubule branching. *Nat Commun* **13**, 5635.

Second decision letter

MS ID#: JOCES/2023/261096

MS TITLE: Microtubule binding of the human HAUS complex is directly controlled by importins and Ran-GTP

AUTHORS: Kseniya Ustinova, Felix Ruhnow, Maria Gili, and Thomas Surrey
ARTICLE TYPE: Research Article

I am happy to tell you that your manuscript has been accepted for publication in Journal of Cell Science, pending standard ethics checks.

Reviewer 1

Advance summary and potential significance to field

The authors have appropriately addressed my previous comments.

Comments for the author

n/a

Reviewer 2

Advance summary and potential significance to field

I am happy to reiterate from my previous review: The HAUS/Augmin complex is an intriguing octameric protein assembly that mediates branched nucleation from existing microtubules. After a

© 2023. Published by The Company of Biologists under the terms of the Creative Commons Attribution License (https://creativecommons.org/licenses/by/4.0/).

flurry of manuscripts from multiple spindle microtubule powerhouses, our understanding of the importance and architecture of the HAUS complex dramatically improved in 2022. However, we need to learn more about the regulation of this crucial complex.

In the current manuscript, Ustinova and colleagues report the regulation HAUS complex by importins and Ran-GTP. In particular, the intrinsically disordered N-terminus of HAUS-8 that the authors find to serve as both a nuclear localization signal/importin binding site and a known microtubule binding motif.
It was previously thought that the nucleation factor TPX2, which might stimulate branched microtubule nucleation, would be the target of importin. However, TPX2 seems to be optional for the process.

The novelty and quality of the work presented are outstanding.
The concentration-dependent effect of importin binding, physiological concentration range, and the
Â‘rescueÂ’ through constitutively active RanQ69L make this a convincing story.

The addition of the newly presented subtilisin experiments fully satisfy my curiosity about the contribution of the HAUS8 binding site to the targeting of the HAUS complex to microtubules.

Comments for the author

I have no additional questions.
The manuscript should be published without delay.

© 2023. Published by The Company of Biologists under the terms of the Creative Commons Attribution License (https://creativecommons.org/licenses/by/4.0/).
